# Vitamin K2 Supplementation in Hospitalised COVID-19 Patients: A Randomised Controlled Trial

**DOI:** 10.3390/jcm13123476

**Published:** 2024-06-14

**Authors:** Margot P. J. Visser, Anton S. M. Dofferhoff, Jody M. W. van den Ouweland, Pim A. de Jong, Pieter Zanen, Henny van Daal, Eline B. Theeuwen, Cornelis Kramers, Rob Janssen, Jona Walk

**Affiliations:** 1 Department of Pulmonary Medicine, Canisius-Wilhelmina Hospital, 6532 SZ Nijmegen, The Netherlands; 2 Department of Internal Medicine, Canisius-Wilhelmina Hospital, 6532 SZ Nijmegen, The Netherlands; 3 Department of Clinical Chemistry, Canisius-Wilhelmina Hospital, 6532 SZ Nijmegen, The Netherlands; 4 Department of Radiology, University Medical Centre Utrecht and Utrecht University, 3584 CX Utrecht, The Netherlands; 5 Department of Pulmonary Diseases, St Antonius Hospital, 3435 CM Nieuwegein, The Netherlands; 6 Department of Pharmacology-Toxicology and Internal Medicine, Radboud University Medical Centre, 6525 GA Nijmegen, The Netherlands

**Keywords:** COVID-19, vitamin K, menaquinone-7, matrix gla protein, dp-ucMGP, desmosine

## Abstract

**Background:** In observational studies, high levels of desphospho-uncarboxylated matrix gla protein (dp-ucMGP) that result from vitamin K deficiency were consistently associated with poor clinical outcomes during COVID-19. Vitamin K-activated matrix gla protein (MGP) is required to protect against elastic fibre degradation, and a deficiency may contribute to pathology. However, intervention trials assessing the effects of vitamin K supplementation in COVID-19 are lacking. **Methods:** This is a single-centre, phase 2, double-blind, randomised, placebo-controlled trial investigating the effects of vitamin K2 supplementation in 40 hospitalised COVID-19 patients requiring supplemental oxygen. Individuals were randomly assigned in a 1:1 ratio to receive 999 mcg of vitamin K2—menaquinone-7 (MK-7)—or a placebo daily until discharge or for a maximum of 14 days. Dp-ucMGP, the rate of elastic fibre degradation quantified by desmosine, and hepatic vitamin K status quantified by PIVKA-II were measured. Grade 3 and 4 adverse events were collected daily. As an exploratory objective, circulating vitamin K2 levels were measured. **Results:** Vitamin K2 was well tolerated and did not increase the number of adverse events. A linear mixed model analysis showed that dp-ucMGP and PIVKA-II decreased significantly in subjects that received supplementation compared to the controls (*p* = 0.008 and *p* = 0.0017, respectively), reflecting improved vitamin K status. The decrease in dp-ucMGP correlated with higher plasma MK-7 levels (*p* = 0.015). No significant effect on desmosine was found (*p* = 0.545). **Conclusions:** These results demonstrate that vitamin K2 supplementation during COVID-19 is safe and decreases dp-ucMGP. However, the current dose of vitamin K2 failed to show a protective effect against elastic fibre degradation.

## 1. Introduction

Despite a decrease in the number of individuals suffering from severe COVID-19, it remains a cause of hospitalisation, particularly for the elderly and individuals with specific comorbidities, underscoring a continued need to elucidate disease pathways and improve treatment strategies. The pathology of severe disease is thought to result from the following two processes: excessive inflammatory response and hypercoagulability [1]. Vitamin K has been implicated in both processes [2,3,4]. It is most commonly known as a co-factor in the activation of clotting factors II, VII, IX and X [5,6]. However, vitamin K is also required to activate anticoagulant proteins C and S, as well as non-coagulation-related factors, such as matrix gla protein (MGP) [5,6,7]. Functional MGP depends on two post-translational modifications, phosphorylation and vitamin K-dependent carboxylation, and is essential to protect elastic fibres against calcification and degradation [6,8,9]. Elastic fibres are fundamental components of dynamic tissues, such as the lungs and arteries [10]. During the first COVID-19 wave in 2020, it was demonstrated that COVID-19 patients had a vitamin K insufficiency, and this correlated with more severe elastic fibre degradation and increased pro-inflammatory cytokine production [11,12,13].

After intestinal absorption, vitamin K is first transported to the liver, where it activates coagulation factors before it is made available to other tissues for the activation of extrahepatic proteins, such as MGP [11]. This means that extrahepatically synthesised vitamin K-dependent proteins can be functionally compromised even while pro-coagulation factors remain intact. This was demonstrated by a study that found increased levels of non-carboxylated (inactive) MGP during COVID-19, while the activation of hepatic vitamin K-dependent procoagulant factor II was less affected [11]. Unlike the procoagulant factors and anticoagulant protein C, approximately 50% of circulating anticoagulant protein S is extrahepatically synthesised, requiring extrahepatic vitamin K-dependent activation [14,15]. Therefore, like MGP [16], the activation of endothelial protein S may be compromised during COVID-19 while pro-coagulant factors remain intact, potentially increasing thrombosis risk [17].

Increasing extrahepatic vitamin K levels during COVID-19 may have favourable effects on both pulmonary damage and thrombosis risk. We conducted a clinical trial to assess the effects of vitamin K supplementation on MGP and elastic fibre degradation in COVID-19 patients.

## 2. Methods

This was a single-centre, phase 2, double-blind, randomised, placebo-controlled trial investigating the effects of oral vitamin K2 supplementation in hospitalised COVID-19 patients. Forty adults with laboratory-confirmed COVID-19 by a Polymerase Chain Reaction (PCR) who required supplemental oxygen were enrolled at the Canisius-Wilhelmina Hospital in Nijmegen, The Netherlands, between 22 February and 15 June 2021. Patients provided written informed consent before participation. The study was approved by the ethical review board CMO Arnhem-Nijmegen on 15 February 2021 (NL74583.091.20) and prospectively registered at ClinicalTrials.gov, identifier NCT04770740. Patients had to be able to safely swallow study medication or have a nasogastric tube through which medication could be administered and also receive a prophylactic or therapeutic dose of low-molecular-weight heparin (LMWH) according to the hospital’s COVID-19 treatment protocols. Individuals were excluded from participation if they were unable to give informed consent, were in need of mechanical ventilation, were haemodialysis dependent, pregnant or had a diagnosed, active malignancy at presentation. Individuals were also excluded when they remained on oral anticoagulant drugs during admission, if they had used vitamin K supplements within 30 days before admission, participated in other intervention studies, or were allergic to the tablet components.

Individuals were randomised in a 1:1 ratio receiving either 999 mcg of dietary supplement vitamin K2—in the form of menaquinone-7 (MK-7)—each day (three tablets of 333 mcg K2VITAL^®^DELTA from Kappa Bioscience AS for oral use) or a matched placebo until discharge, with a maximum treatment duration of 14 days. Patient recruitment was conducted at the hospital by the clinical investigator. It was a double-blind study where patients, treating physicians, investigators and other collaborators were blinded to the identity of study groups until all patients completed primary endpoint assessments. The allocation order was determined using a treatment list randomly generated in Excel (Excel 2016, Microsoft, Redmond, WA, USA). Our hospital’s pharmacy employees who were not involved in patient care were responsible for the correct execution of randomisation and treatment number assignment to either the intervention or placebo group. Subsequently, Kappa Bioscience AS labelled the intervention and placebo boxes according to the generated randomisation list. Patients included in the study were assigned to the lowest treatment number available at the trial site (ranging from 0 to 40). Treatment numbers were registered on the drug accountability log form and eCRF (electronic Case Report Form). The sample size was based on the estimated effect of vitamin K2 on plasma desmosine, considering a difference of 10% as clinically significant (with α = 0.05 and β = 0.20). In a study of 135 COVID-19 patients’ plasma, desmosine had a log-normal distribution, with a mean ln of 5.99 with a standard deviation of 0.603 [11]. The effects of vitamin K2 on this outcome have never been studied; however, we consider a difference in ln (plasma DES) of 10% to be clinically significant. An independent samples *t*-test (with α = 0.05 and β = 0.20) would be statistically significant between two groups of 16 persons (and a total study population of 32 persons). Based on these two outcomes, the sample size of 40 persons allows for a 10% loss to follow-up for the per-protocol analysis.

An independent Safety Monitoring Committee (SMC) was appointed to perform an interim analysis focusing on the identification of any potential serious adverse effects. According to local hospitals and National Guidelines for the treatment of COVID-19, applicable at the time of inclusion, patients who required supplemental oxygen were treated with anti-inflammatory drugs like dexamethasone and/or tocilizumab.

### 2.1. Outcome Measures

Dp-ucMGP, desmosine (a marker for elastic fibre degradation), proteins induced by vitamin K absence or antagonist-II (PIVKA-II), and vitamin K2 MK-7 were determined in the blood samples before and during treatment with either vitamin K or a placebo three times a week until discharge. Grade 3 and 4 adverse events were collected daily (according to the NCI Common Terminology Criteria for Adverse Events version 5). Coagulation or bleeding events were regarded as adverse events of specific significance. Mortality and serious adverse events were followed up until day 28.

In addition, interleukin-6 (IL-6) and D-dimer were determined, and changes in disease severity on unenhanced low-dose chest Computed Tomography (CT) were included as an exploratory outcome. Two CT scans were made, first at admission and second, on day 5 or at discharge, whichever occurred first. The severity of COVID-19 pneumonia was quantified and expressed as the volume percentage of lung involvement on an unenhanced low-dose chest CT by a specialised chest radiologist at University Medical Centre Utrecht (UMCU), Utrecht, The Netherlands.

### 2.2. Blood Samples and Laboratory Assays

The levels of non-carboxylated vitamin K-dependent proteins in the blood circulation are a more accurate measurement of overall vitamin K status than the direct measurement of circulating vitamin K levels due to differences in bioavailability, tissue distribution and the half-life time between the two naturally occurring vitamin K forms (vitamin K1 and K2) [18]. Dp-ucMGP and PIVKA-II inversely correlate with extrahepatic and hepatic vitamin K status [19,20]. High dp-ucMGP/PIVKA-II levels reflect low vitamin K status and vice versa. Dp-ucMGP values > 500 pmol/L reflect extrahepatic vitamin K deficiency [21,22]. PIVKA-II levels were compared with the PIVKA-II concentrations of healthy individuals presented in the Roche Diagnostics (Elecsys^®^ PIVKA-II) protocol. The median of PIVKA-II concentration in 811 healthy adults was 19.7 ng/mL with a 95th percentile of 28.4 ng/mL.

Desmosine and isodesmosine fractions were measured separately by liquid chromatography–tandem mass spectrometry using deuterium-labelled desmosine as an internal standard [23,24]. Age-dependent desmosine values of healthy never-smokers (a control group of Huang et al.) were used as the reference (desmosine value of 219 ng/L) [25]. Circulating vitamin K2 MK-7 plasma levels were measured by liquid chromatography with tandem mass spectrometry (LC-MS/MS) with a lower limit of quantification of 0.52 ng/mL. A detailed description of blood sampling processes and laboratory assays can be found in the trial protocol, which is provided as Supplementary Material File S1.

### 2.3. Statistical Analysis

Statistical analysis was performed using SPSS (version 24, IBM SPSS Statistics for Windows, Europe) and GraphPad Prism software (version 5.03, GraphPad Software Inc., San Diego, CA, USA). Statistical analysis was performed using intention-to-treat (ITT) analysis. Results were displayed as the mean (SD) for continuous normally distributed variables and median (IQR) for not normally distributed variables. Categorical variables were presented as frequencies (N) and percentages (%). To compare patient characteristics between the intervention and control group at the baseline, two-sample *t*-tests (α = 0.05) for normally distributed data and a Mann–Whitney test for not normally distributed data were used. In addition, Fisher’s exact test was performed to compare categorical data.

To account for the wide distribution and variation in the number of time points per subject, a linear mixed model was used to analyse the changes over time of dp-ucMGP, PIVKA-II, desmosine and the circulating vitamin K2 MK-7 plasma levels in treatment versus control patients. The data were collected on consecutive time points within subjects and are, thus, correlated. The standard approach to this longitudinal design is a mixed model analysis where time is inserted into the analytical model as a random parameter. This approach used a random intercept and slope. Analytical model comparisons were performed using Akaike’s Information Criterion (AIC). The analytical model used age at the start, Body Mass Index (BMI), gender and treatment as explanatory parameters. The model was expanded by the interaction between treatment and time. The latter term depicts whether the time course of the outcome parameters (dp-ucMGP, PIVKA-II, desmosine) differs between the intervention and control group as follows: when a steeper decline/rise is present in one of the two groups, the interaction turns out to be significant. For the circulating vitamin K2 MK-7 plasma level analysis, only data from the intervention group were used because all values in the control group were below the limit of quantification. The outcome of the procedure presents regression equations, which were transferred to Excel for further calculations and graph construction. The validity of the mixed model analysis outcomes was assessed via the distribution of the residual errors. A histogram and Q-Q plot inspection assessed the normal distribution of these residuals. All biological data were shown to be ln-normally distributed and needed ln-transformation, which lowered the AIC considerably. The outcome of any analysis delivers differences between ln-transformed data, which were subsequently back-transformed via e-power to obtain ratios. *p* < 0.05 is considered significant.

## 3. Results

**Figure 1:**
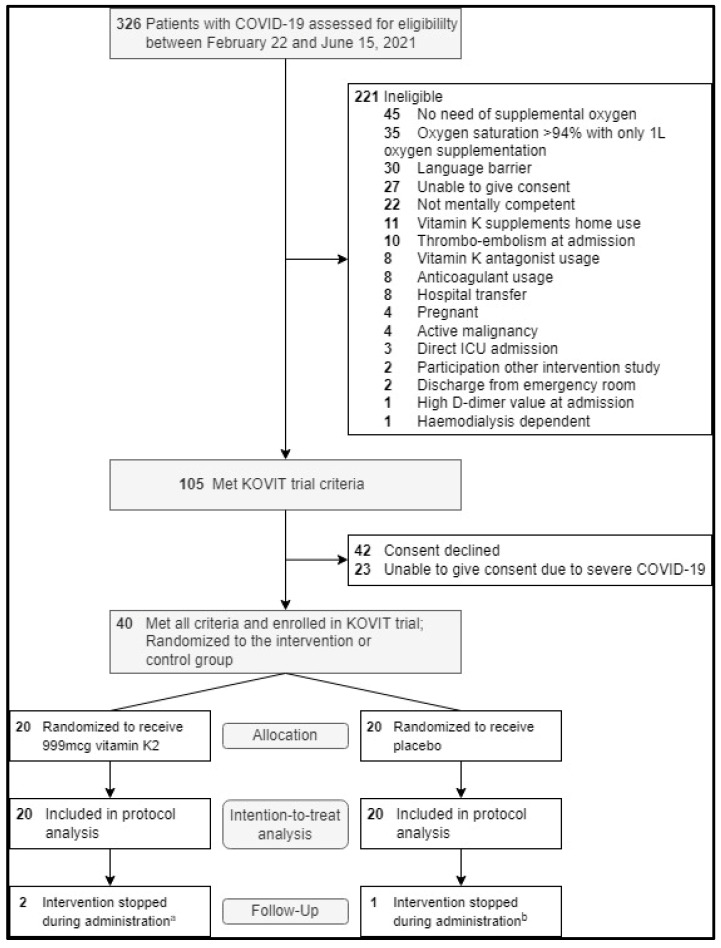
Flowchart of KOVIT trial. COVID-19: Coronavirus 2019; ICU: Intensive Care Unit. ^a^ One patient withdrew from participation on study day 2, and one patient withdrew from participation on study day 5. ^b^ Patient withdrew from participation on day 9 of the study intervention.

All 40 patients were included in the intention-to-treat analysis. The last patient visit was on 12 July 2021. Three patients withdrew from participation during the intervention phase of the study: one after the first study dose because of the repeated failure of peripheral blood sampling and two due to the burden of extra blood sampling and/or a second CT scan. Two patients who were admitted to the ICU during the intervention were transferred to another hospital due to space limitations. The intervention stopped after they left the hospital. Mortality and serious adverse events were followed until day 28 in all patients, including those who withdrew or who were transferred. Details about the follow-up and a flowchart of the KOVIT trial are shown in Figure 1. Baseline characteristics concerning age, gender, and BMI were similarly distributed across groups and typical for patients requiring hospital admission for COVID-19; patient characteristics are shown in Table 1.

All individuals were included within 24 h after presentation to the emergency department. The median duration of hospital admission was 5 days in patients receiving vitamin K2 versus 6 days in those from the control group. Disease progression and additional treatments with antibiotics, dexamethasone or tocilizumab during hospital admission are shown in Table 2.

### 3.1. Outcome

**Figure 2:**
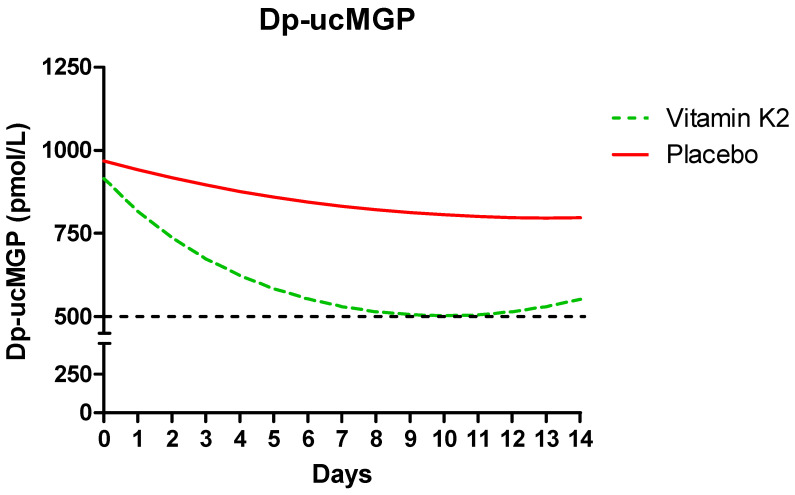
Visual representation of decline in desphospho-uncarboxylated matrix gla protein (dp-ucMGP) in the intervention (*n* = 20) and control groups (*n* = 20). The regression equation of the mixed model analysis was transferred to Excel, and subsequently, relevant choices for age at the start, BMI, gender, treatment and study day were inserted, resulting in an outcome for dp-ucMGP used for visual representation with a significant steep decline in the intervention group (*p* = 0.008).

To account for the wide distribution at baseline, a linear mixed model was used to analyse changes in plasma dp-ucMGP, desmosine levels and PIVKA-II over time between the two groups. Age at the start, BMI and gender did not significantly differ in any of the analyses and were removed from the analytical model. Dp-ucMGP levels were elevated (>500 pmol/L) in 97.5% (39/40) of patients at admission, indicating functional vitamin K insufficiency. Dp-ucMGP levels significantly decreased over time in this study (*p* < 0.001) (Table 3). In the intervention group, there was a significant decline in dp-ucMGP; it decreased by a factor of 0.930 each day compared to a factor of 1.004 in the control group. The difference is significant (*p* = 0.008) (Table 3). Figure 2 shows a visual representation of the steeper decline in the intervention group compared to the control group. In the intervention group, there was a significant effect of increasing circulating vitamin K2 MK-7 plasma levels on dp-ucMGP; for each unit increase in MK-7 levels, dp-ucMGP decreased by a factor 0.996 (*p* = 0.015) (Table 3). Of note, in two patients admitted for more than two weeks, dp-ucMGP swiftly increased after supplementation was discontinued after 14 days.

Desmosine levels were not increased at baseline compared to historical never-smoking controls [25]. A change in desmosine in response to time was not observed (*p* = 0.125), nor was there a different time * treatment interaction (*p* = 0.545) (Table 3).

At admission, PIVKA-II levels were above 30 ng/mL in all participants and severely elevated in 27.5% of patients (severely elevated was defined as >79 ng/mL; *n* = 11/40). A change in PIVKA-II levels in response to time was observed (*p* < 0.001) (Table 3). In the intervention group, there was a significantly steeper decline; PIVKA-II decreased by a factor of 0.917 each day, compared to a factor of 0.987 in the control group. This difference was significant (*p* = 0.0017) (Table 3). In the intervention group, there was a significant effect of increasing vitamin K2 MK-7 plasma levels on PIKA-II; for each unit increase in MK-7, PIVKA-II decreased by a factor of 0.994 (*p* < 0.001) (Table 3).

**Figure 3:**
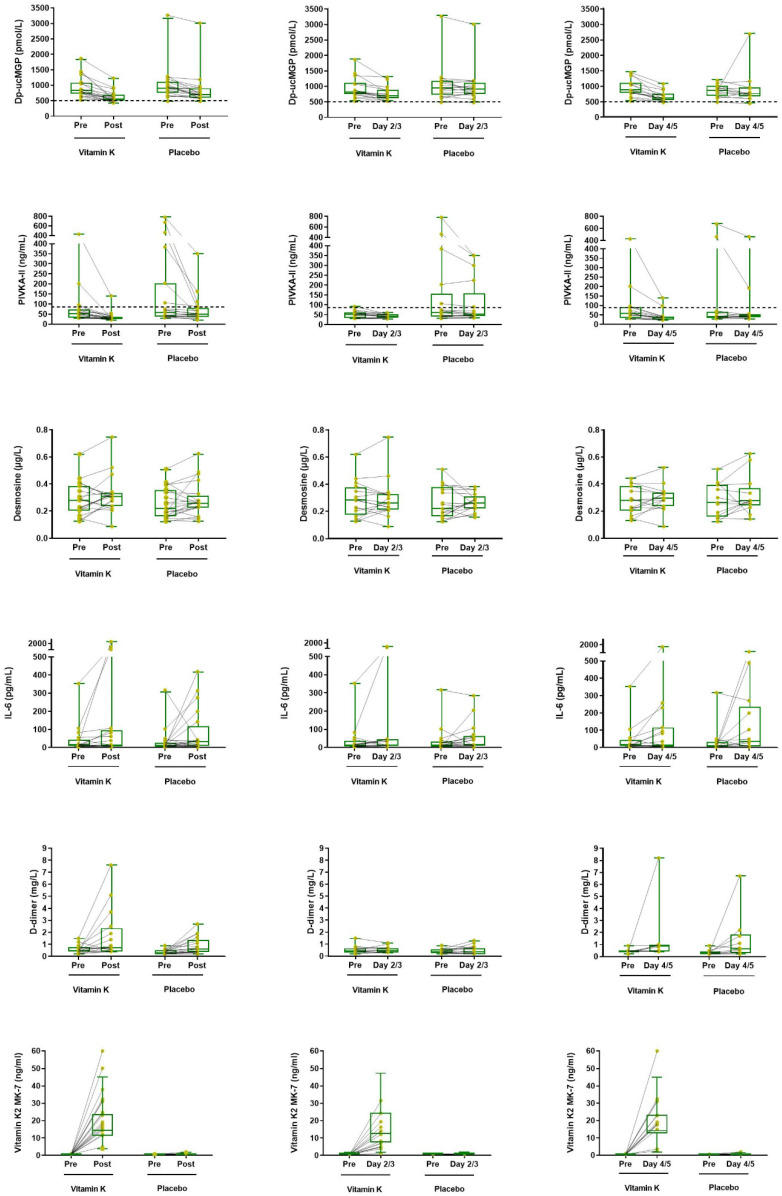
Desphospho-uncarboxylated matrix gla protein (dp-ucMGP), protein induced by vitamin K absence or antagonist-II (PIVKA-II), desmosine, interleukin-6 (IL-6), D-dimer and circulating vitamin K2 menaquinone-7 (MK-7) plasma levels of patients receiving either vitamin K2 or placebo. Figures show a combined before–after plot and boxplot of measured outcome parameters. Dashed lines indicate upper levels of normal ranges. All parameters were measured at different points in time during hospital admission. ‘Pre’ is the first blood sampling after inclusion and before the start of tablet supplementation. ‘Post’ is the last blood sampling point of individual patients; this day can vary between 2 and 14 days. During that time period, patients were admitted and received our supplements. ‘Day 2/3’ and ‘Day 4/5’ are time points in which the next blood sampling was taken. Blood was sampled every other day (on Monday, Wednesday, Friday or Saturday/Sunday).

No significant reduction in IL-6 levels or D-dimer was found when comparing groups. Figure 3 shows all parameters at different time points.

In total, 40 CT scans were made at inclusion, and 33 CT scans were made on day 5 or earlier (18 CT scans were made on exactly day 5). Not all patients were able to undergo a second CT scan. There was no significant difference between the first and second CT scans in the volume of ground glass and consolidation when comparing groups (*p* = 0.747).

### 3.2. Adverse Events

Vitamin K2 was well tolerated, and no difference in thrombotic complications or other safety issues were identified between the groups. No patients suffered from gastro-intestinal symptoms, and antibiotic use was equally distributed between the groups (*n* = 10 in the placebo group; *n* = 11 in the vitamin K2 group). There were no significant between-group differences in serious adverse events and/or thrombotic complications. Five patients in the intervention group, compared to six patients in the control group, incurred one or two serious adverse events or coagulation-related events. Two patients suffered from a thrombotic event (pulmonary embolism *n* = 2) in the intervention group compared to four patients suffering from a thrombotic event (pulmonary embolism *n* = 3; deep vein thrombosis *n* = 1) in the control group. One patient in the intervention group died six days after study initiation due to severe respiratory insufficiency combined with an ST-elevation myocardial infarction. Of the patients in the vitamin K2 group, three patients were transferred to the ICU compared to one patient receiving a placebo. Details about the disease course of these patients are shown in Table 2.

## 4. Discussion

Here, we demonstrate that the oral administration of vitamin K2 partially corrects vitamin K deficiency in hospitalised COVID-19 patients. The decrease in dp-ucMGP was greater in those with higher plasma vitamin K2 (MK-7) levels. The intervention was well-tolerated, and there were no relevant differences in adverse events between groups. Vitamin K2 did not affect the rate of elastic fibre degradation.

Multiple observational studies have found high levels of dp-ucMGP in hospitalised COVID-19 patients, indicating significant vitamin K deficiency in the extrahepatic compartment [11,26,27,28]. Increased dp-ucMGP was consistently associated with the risk of severe disease and poor clinical outcome [11,26,27,28]. Similarly, other recent studies found that vitamin K2 subtype menaquinone-7 was decreased in hospitalised COVID-19 patients [29] and that high PIVKA-II was associated with ICU admission and death in COVID-19 patients [30]. However, the effect of vitamin K supplementation in COVID-19 has not been studied until now. The current trial confirms previous findings of increased dp-ucMGP in COVID-19 patients and demonstrates for the first time that dp-ucMGP can be corrected with vitamin K supplementation in these patients.

A decrease in dp-ucMGP levels below 500 pmol/L—arbitrarily regarded as the upper limit of normal—was not achieved with the vitamin K2 dose used in this trial despite using doses that have previously been demonstrated to be sufficient for this decrease in dp-ucMGP in healthy individuals [31,32,33,34]. None of the study participants had gastro-intestinal symptoms, such as diarrhoea or vomiting, that could have reduced absorption. Concomitant antibiotic use can affect the intestinal absorption of vitamin K. However, we consider this unlikely to be a major determinant as there was a swift increase in circulating MK-7 in all patients after vitamin K2 administration, irrespective of antibiotic use. Alternatively, the complete activation of dp-ucMGP may theoretically be impeded by limitations in gamma-glutamyl carboxylase activity, but this idea was refuted by the observation of a rapid decrease in PIVKA-II in all patients, indicating effective carboxylation. A more plausible explanation is that there is an ongoing depletion of vitamin K during SARS-CoV-2 infection, especially in the extrahepatic compartment. Indeed, indirect evidence shows that severe inflammatory responses correlate with increased dp-ucMGP and may represent an important contributor to extrahepatic vitamin K depletion [35,36,37]. The observed immediate increase in dp-ucMGP and decrease in circulating MK-7 after terminating vitamin K2 administration in patients with ongoing infection supports this hypothesis. Considering the overwhelming inflammation in severe COVID-19, it is possible that far higher doses are required in COVID-19 patients to compensate for the excessive vitamin K utilisation. The dose–response relationship between plasma MK-7 and dp-ucMGP levels also suggests that the dose was insufficient to achieve optimal extrahepatic carboxylation. Furthermore, we observed that PIVKA-II—reflecting hepatic rather than extrahepatic vitamin K status—was increased in a large number of patients at baseline. PIVKA-II decreased faster than dp-ucMGP during vitamin K supplementation. This is compatible with the preferential distribution of vitamin K to the liver in states of limited availability (Figure 3) [38]. This supports the need for higher doses of oral vitamin K2 in future COVID-19 trials for the more effective restoration of extrahepatic stores.

Vitamin K may also impact the progression of COVID-19 in other ways. A recent study reported on the potential protective effects of vitamin K3—a synthetic form of vitamin K—on SARS-CoV-2-induced inflammation and cell damage [39]. The authors demonstrated that vitamin K3 might have beneficial effects on SARS-CoV-2 E protein-induced-cell death (pyroptosis) and reduced N protein-induced monocyte adhesion [39]. Staufer et al. suggest that vitamin K may directly bind the SARS-CoV-2 free fatty acid-binding pocket (FABP), potentially impacting infectivity [40].

No adverse effects have been reported for individuals consuming high doses of vitamin K, and no tolerable upper intake level has been established by authorities [41]. Nevertheless, concerns have been raised about the safety of vitamin K administration, specifically in COVID-19, due to the pro-thrombotic nature of the disease. This was the first trial to investigate the safety of vitamin K supplementation in COVID-19. Similar to other conditions [42], we found no toxicity or other adverse effects during our treatment. Furthermore, there was no increase in thrombotic complications or other safety issues in the vitamin K-treated group.

Accelerated systemic elastic fibre degradation—quantified with the biomarker desmosine—has been demonstrated in COVID-19 and was related to poor clinical outcomes [11]. Based on the known association between desmosine and dp-ucMGP [20], we chose desmosine as a surrogate endpoint of our study. Although we found a significant decrease in dp-ucMGP, we did not observe a reducing effect of vitamin K2 supplementation on desmosine levels. However, desmosine levels were not elevated at baseline compared with age-adjusted historical controls [25]. This is in contrast to our earlier findings in the first COVID-19 wave in which we demonstrated elevated dp-ucMGP and desmosine levels correlating with poor clinical outcome [11]. This difference may indicate a study population with less severe disease or may be due to improved COVID-19 treatment protocols.

Furthermore, no effect of vitamin K2 supplementation was found on pneumonia severity on the CT scan nor on circulating IL-6. As the vitamin K2 therapy in this study did not result in dp-ucMGP levels below 500 pmol/L, it is still possible that increased dosages could protect against inflammation or lung damage in COVID-19. Future studies should be conducted either using a higher dosage of vitamin K2 or starting supplementation earlier in the course of the disease, for instance, prior to hospital admission.

### Limitations

This study was of high quality, given the placebo-controlled, double-blinded design. However, some limitations need to be addressed. The study was not powered to assess whether vitamin K supplementation could have positive effects on the clinical outcome of COVID-19. However, there are no obstacles to conducting a larger study considering the demonstrated lack of safety concerns of the intervention in this pilot study.

Another study limitation was that COVID-19 patients who were more severely affected and admitted to our wards were less likely to be included because the disease burden was usually too high in these individuals to fully understand the study information and decide about participation. This may also contribute to the fact that baseline dp-ucMGP, desmosine and IL-6 levels were lower in the current study population compared to our previous observational study.

## 5. Conclusions

We demonstrated that vitamin K2 administration lowers dp-ucMGP as a potentially modifiable risk factor for severe COVID-19 and appears safe with no increased thromboembolic complications. Larger trials using a higher dosage are warranted to evaluate whether vitamin K2 can improve clinical outcomes in COVID-19.

## Figures and Tables

**Table 1:** Participant characteristics at baseline.

Characteristic	Vitamin K2 999 mcg (*n* = 20)	Placebo (*n* = 20)	*p*-Value
Age, mean (SD)	59.8 (10.4)	56.2 (8.3)	0.343
Male (%)	13 (65)	15 (75)	0.731
BMI, kg/m^2^, (SD)	30 (4.8)	28.7 (3.0)	0.342
**Pre-existing conditions** **No. (%) of participants**			
Hypertension	4 (20)	3 (15)	0.999
Diabetes mellitus	2 (10)	5 (25)	0.408
Cardiac or cardiovascular disease	4 (20)	1 (5)	0.342
Asthma/COPD	5 (25)	3 (15)	0.695
Other respiratory disease	2 (10)	2 (10)	0.999
Immunocompromised	0	1 (5)	0.999
Platelet aggregation inhibitor use	5 (25)	1 (5)	0.182
First COVID-19 vaccine	1 (5)	3 (15)	0.605
Current smoker	0	0	0.999
Former smoker	12 (60)	7 (35)	0.205
**Acute physiology and laboratory values** No. Number of participants			
**PO_2_, mm/Hg, No.**	19	20	
Median (LQ, UQ)	55 (52, 58)	58.5 (50.8, 64.8)	0.147
**Lymphocytes, ×10^9^/L, No.**	20	20	
Median (LQ, UQ)	0.95 (0.63, 1.25)	0.85 (0.60, 1.20)	0.341
**CRP, mg/L, No.**	20	20	
Median (LQ, UQ)	89.5, (65.5, 115.8)	108.5 (51.5, 139.8)	0.743
**LDH, U/L, No.**	20	20	
Median (LQ, UQ)	394.5 (322.3, 479)	397.5 (293.5, 524.3)	0.963
**Creatinine, µmol/L, No.**	20	20	
Median (LQ, UQ)	81 (68, 91.8)	68.5 (61.5, 90.5)	0.324
**Dp-ucMGP, pmol/L, No.**	20	20	
Median (LQ, UQ)	836.7 (734, 1092)	915.1 (765.5, 1122)	0.678
**PIVKA-II, ng/mL, No.**	20	20	
Median (LQ, UQ)	55.1 (35, 87.2)	53.6 (39.2, 179.3)	0.565
**Desmosine, µg/L, No.**	20	20	
Median (LQ, UQ)	0.281 (0.210, 0.380)	0.219 (0.160, 0.355)	0.344
**IL-6, pg/mL, No.**	20	20	
Median (LQ, UQ)	15.8 (7.90, 37.6)	12.31 (3.63, 27.51)	0.370
**D-dimer, mg/L, No.**	18	19	
Median (LQ, UQ)	0.45 (0.3, 0.7)	0.3 (0.2, 0.5)	0.120

SD: Standard deviation; LQ: lower quartile; UQ: upper quartile; BMI: Body Mass Index; COPD: chronic obstructive pulmonary disease; COVID-19: Coronavirus 2019; CRP: C-reactive protein; LDH: lactate dehydrogenase; Dp-ucMGP: desphospho-uncarboxylated matrix gla protein; PIVKA-II: protein induced by vitamin K absence or antagonist-II; IL-6: interleukin-6.

**Table 2:** Disease progression, additional treatments, serious adverse events and coagulation-related events until study day 28.

	Vitamin K2, 999 mcg (*n* = 20)	Placebo (*n* = 20)	*p*-Value
Outcome/treatment			
Duration of hospital admission, days, median (LQ, UQ)	5 (4, 10)	6 (4, 11)	0.941
Number of days in trial, days, median (LQ, UQ)	5 (4, 10)	6 (4, 11)	0.941
Suspicion of bacterial infection in patients treated with antibiotics, No. (%)	11 (55)	10 (50)	0.999
Number of patients treated with dexamethasone, No (%)	19 (95)	20 (100)	0.999
Number of patients treated with tocilizumab, No (%)	5 (25)	6 (30)	0.999
Adverse events, No. (%)			
Number of patients with relevant adverse events	5 (25)	6 (30)	0.999
High-flow nasal cannula	1 (5) ^1^	0	0.999
ICU admission with mechanical ventilation	3 (15) ^2,3^	1 (5) ^4^	0.605
Deceased	1 (5)	0	0.999
Deep vein thrombosis	0	1 (5)	0.999
Pulmonary embolism	2 (10) ^2,3^	3 (15) ^4,5^	0.999
Increase in D-dimer	1 (5) ^1^	2 (10)	0.999
Rectal haemorrhage	0	1 (5) ^5^	0.999

LQ: Lower quartile; UQ: upper quartile; ICU: Intensive Care Unit. ^1^ Patient: increased D-dimer was measured on study day 5, and progression of respiratory insufficiency with the need for high-flow nasal oxygen supplementation on study day 6 was determined. After study day 6, vitamin K2 supplementation stopped because the patient withdrew from participation. The patient recovered. ^2^ Patient: progression of respiratory insufficiency with the need for mechanical ventilation in the ICU occurred on study day 3. On study day 6, a pulmonary embolism was diagnosed using CT angiography. Vitamin K2 supplementation continued during those events. The patient recovered. ^3^ Patient: progression of respiratory insufficiency with the need for mechanical ventilation in the ICU occurred on study day 2. On study day 7, a pulmonary embolism was diagnosed using CT angiography. Vitamin K2 supplementation continued during these events. The patient recovered. ^4^ Patient: two events occurred within the same patient receiving a placebo. ^5^ Patient: two events occurred within the same patient receiving a placebo.

**Table 3:** Linear mixed model analysis summary table.

	*p*-Value	Daily Change (as Ratio)	95% Confidence Interval (CI)
**Dp-ucMGP, pmol/L**			
Time effect	<0.001	0.930	(0.906–0.954)
Time * treatment interaction	0.008	1.079	(1.026–1.136)
Change in the intervention group		0.930	
Change in the control group		1.004	
**PIVKA-II, ng/mL**			
Time effect	<0.001	0.917	(0.890–0.944)
Time * treatment interaction	0.0017	1.077	(1.015–1.142)
Change in the intervention group		0.917	
Change in the control group		0.987	
**Desmosine, µg/L**			
Time effect	0.125	1.019	(0.995–1.043)
Time * treatment interaction	0.545	0.986	(0.941–1.033)
	***p*** **-Value**	**Change** **(as ratio)**	**95% Confidence Interval (CI)**
**Dp-ucMGP, pmol/L**			
Unit increase vitamin K2 MK-7 plasma level	0.015	0.996	(0.994–0.997)
**PIVKA-II, ng/mL**			
Unit increase vitamin K2 MK-7 plasma level	<0.001	0.994	(0.990–0.997)

Dp-ucMGP: desphospho-uncarboxylated matrix gla protein; PIVKA-II: a protein induced by vitamin K absence or antagonist-II. Time effect: changes in dp-ucMGP, PIVKA-II and desmosine in response to time in both groups. Time * treatment interaction: effect of time * treatment interaction on changes in dp-ucMGP, PIVKA-II and desmosine. Change in the control/intervention group: the daily changes within each group are listed for dp-ucMGP and PIVKA-II. Changes in the control group are based on the effects of both time and time * treatment interaction. The intervention group’s (=reference) daily change is equal to the time effect. A unit increase in vitamin K2 MK-7 plasma level: changes in dp-ucMGP/PIVKA-II elicited by a unit increase in circulating vitamin K2 MK-7 plasma levels. Estimates constitute daily changes (as a ratio) and are listed with their 95% confidence interval (CI).

## Data Availability

The dataset used during the current study is available from the corresponding author upon reasonable request.

## Supplementary Materials

The following supporting information can be downloaded at https://www.mdpi.com/article/10.3390/jcm13123476/s1, File S1. Protocol for KOVIT trial.

## Abbreviations

COVID-19: Coronavirus Disease 2019; dp-uc, desphospho-uncarboxylated; MGP, matrix gla protein; SARS-CoV, severe acute respiratory syndrome coronavirus; MK-7, menaquinone-7; PIVKA-II, protein induced by vitamin K absence or antagonist-II; PCR, Polymerase Chain Reaction; LMWH, low-molecular-weight heparin; eCRF, electronic Case Report Form; SMC, Safety Monitoring Committee; IL-6, interleukin-6; CT, Computed Tomography; LC-MS/MS, liquid chromatography with tandem mass spectrometry; ITT, intention to treat; AIC, Akaike’s Information Criterion; BMI, Body Mass Index; ICU, Intensive Care Unit.
