# Supplementary material for: Vitamin K2 Supplementation in Hospitalised COVID-19 Patients: A Randomised Controlled Trial
Source: J Clin Med. 2024 Jun 14;13(12):3476. doi: 10.3390/jcm13123476 (PMC11205124; doi:10.3390/jcm13123476)
Supplement: Supplementary file 1 [file jcm-13-03476-s001.zip › jcm-3018092-supplementary.pdf]

RESEARCH PROTOCOL

KOVIT trial

A phase 2, double blind, randomized, placebo controlled clinical trial to investigate the safety and effects of oral vitamin K2 supplementation in COVID-19

PROTOCOL TITLE:

A phase 2, double blind, randomized, placebo controlled clinical trial to investigate the safety and effects of oral vitamin K2 supplementation in COVID-19 disease progression.

**Protocol ID**	**NL74583.091.20**
**Short title**	**KOVIT**
**Version**	**4.0**
**Date**	**23 March 2021**
**Coordinating investigator and project leader**	**Canisius Wilhelmina Ziekenhuis**<br>Department of Internal Medicine<br>Weg door jonkerbos 100<br>6532 SZ Nijmegen<br>**Dr. Jona Walk, MD**<br>[redacted]
**Sponsor**<br>**(In Dutch: verrichter)**	**Canisius Wilhelmina Ziekenhuis (CWZ)**<br>Department of Internal Medicine<br>Weg door jonkerbos 100<br>6532 SZ Nijmegen<br>The Netherlands
**Principal investigator**	**Canisius Wilhelmina Ziekenhuis**<br>Department of Internal Medicine<br>Weg door jonkerbos 100<br>6532 SZ Nijmegen<br>**Dr. Ton Dofferhoff, MD, PhD**<br>[redacted]
**Clinical investigator**	**Canisius Wilhelmina Ziekenhuis**<br>Department of Pulmonary Medicine<br>Weg door jonkerbos 100<br>6532 SZ Nijmegen

	**Margot Visser, MD**<br>[redacted]
**Scientific advisors**	**Canisius Wilhelmina Ziekenhuis**<br>Department of Internal Medicine<br>Weg door jonkerbos 100<br>6532 SZ Nijmegen<br>**Dr. Rob Janssen, MD**<br>[redacted]<br><br>**Radboud university medical center**<br>Department of Internal Medicine<br>Geert Grooteplein 10<br>6500 HB Nijmegen<br>**Prof. Dr. Kees Kramers, MD**<br>[redacted]
**Biological evaluators**	**Canisius Wilhelmina Ziekenhuis**<br>Department of Laboratory Science<br>Weg door jonkerbos 100<br>6532 SZ Nijmegen<br>**Dr. Jody M.W. van den Ouweland**<br>[redacted]<br><br>[redacted]
**Subsidising parties**	**Canisius Wilhelmina Ziekenhuis**<br><br>**Kappa Biosciences AS**
**Independent expert**	[redacted]

	[redacted]
**Laboratory sites**	**Canisius Wilhelmina Ziekenhuis**<br>Clinical Chemistry Laboratory<br>Weg door jonkerbos 100<br>6532 SZ Nijmegen
**Pharmacy**	**Canisius Wilhelmina Ziekenhuis**<br>Clinical Pharmacy<br>Weg door jonkerbos 100<br>6532 SZ Nijmegen

PROTOCOL SIGNATURE SHEET

Name	Signature	Date
[redacted]		
[redacted]		

TABLE OF CONTENTS

1. INTRODUCTION AND RATIONALE 16
1.1 Background 16
1.2 Rationale for the current study 18
2. OBJECTIVES 21
3. STUDY DESIGN 22
4. STUDY POPULATION 23
4.1 Population (base) 23
4.2 Inclusion criteria 23
4.3 Exclusion criteria 23
4.4 Sample size calculation 24
5. TREATMENT OF SUBJECTS 25
5.1 Investigational product 25
5.2 Escape medication 25
6. INVESTIGATIONAL PRODUCT 26
6.1 Name and description of investigational product(s) 26
6.2 Summary of findings from non-clinical studies 28
6.3 Summary of findings from clinical studies 29
6.4 Summary of known and potential risks and benefits 31
6.5 Description and justification of route of administration and dosage 32
6.6 Dosages, dosage modifications and method of administration 32
6.7 Preparation and labelling of Investigational Product 32
6.8 Drug accountability 32
7. NON-INVESTIGATIONAL PRODUCT 34
8. METHODS 36
8.1 Study parameters/endpoints 36
8.1.1 Main study parameters/endpoints 36
8.1.2 Secondary study parameters/endpoints 36
8.1.3 Exploratory study parameters 36
8.2 Randomisation, blinding and treatment allocation 37
8.3 Study procedures 38
8.3.1 Baseline screening 38
8.3.2 Study medication 38
8.3.3 Follow-up of adverse events 38
8.3.4 Vital signs 38
8.3.5 Clinical laboratory measurements 39
8.3.6 Dp-ucMGP 39
8.3.7 PIVKA-II 39
8.3.8 Desmosine 39
8.3.9 Exploratory laboratory measurements 40
8.3.10 Chest Computer Tomography (CT) scans 40

8.3.11 Vitamin K epoxide reductase complex subunit 1 (VKORC-1) gene polymorphisms 41
8.3.12 Study flow chart 42
8.4 Withdrawal of individual subjects 43
8.4.1 Specific criteria for withdrawal 43
8.5 Replacement of individual subjects after withdrawal 43
8.6 Follow-up of subjects withdrawn from treatment 44
8.7 Premature termination of the study 44
9. SAFETY REPORTING 45
9.1 Temporary halt for reasons of subject safety 45
9.2 AEs and SAEs 45
9.2.1 Adverse events (AEs) 45
9.2.2 Serious adverse events (SAEs) 46
9.3 Annual safety report 46
9.4 Follow-up of (serious) adverse events 47
9.4.1 (Serious) adverse event data collection 47
9.4.2 Assessment of causality 47
9.4.3 Follow-up of (serious) adverse events 48
9.5 Safety Monitoring Committee 48
9.5.1 Review of safety data by the safety monitor and SMC 48
9.5.2 Safety stopping rules 49
10. STATISTICAL ANALYSIS 50
10.1 Primary study parameters 50
10.2 Secondary and exploratory study parameters 50
10.3 Interim analysis 50
11. ETHICAL CONSIDERATIONS 51
11.1 Regulation statement 51
11.2 Recruitment and consent 51
11.3 Benefits and risks assessment, group relatedness 52
11.4 Compensation for injury 53
12. ADMINISTRATIVE ASPECTS, MONITORING AND PUBLICATION 54
12.1 Handling and storage of data and documents 54
12.2 Case Report Forms 54
12.3 Subject confidentiality 54
12.4 Filing of essential documents 55
12.5 Storage of samples 55
12.6 Monitoring and Quality Assurance 55
12.7 Amendments 56
12.8 Annual progress report 56
12.9 Temporary halt and (prematurely) end of study report 57
12.10 Public disclosure and publication policy 57
13. STRUCTURED RISK ANALYSIS 58
13.1 Potential issues of concern 58

13.2 Synthesis 60
14. REFERENCES 62

LIST OF ABBREVIATIONS AND RELEVANT DEFINITIONS

ABR	General Assessment and Registration form (ABR form), the application form that is required for submission to the accredited Ethics Committee; in Dutch: Algemeen Beoordelings- en Registratieformulier (ABR-formulier)
AE	Adverse Event
AR	Adverse Reaction
CA	Competent Authority
CCMO	Central Committee on Research Involving Human Subjects; in Dutch: Centrale Commissie Mensgebonden Onderzoek
COVID-19	Coronavirus Disease-19
CRP	C reactive Protein
CT	Computer Tomography
CV	Curriculum Vitae
CWZ	Canisius Wilhelmina Ziekenhuis
Dp-ucMGP	Dephosphorylated-uncarboxylated Matrix Gla Protein
EU	European Union
EudraCT	European drug regulatory affairs Clinical Trials
GCP	Good Clinical Practice
GDPR	General Data Protection Regulation; in Dutch: Algemene Verordening Gegevensbescherming (AVG)
IB	Investigator’s Brochure
IC	Informed Consent
ICU	Intensive Care Unit
IL	Interleukin
IMP	Investigational Medicinal Product
IMPD	Investigational Medicinal Product Dossier
LMWH	Low Molecular weight heparin
METC	Medical research ethics committee (MREC); in Dutch: medisch-ethische toetsingscommissie (METC)
MGP	Matrix Gla Protein
MK-7	Menaquinone-7
MMP	Matrix Maleoproteinases
PCR	Polymerase Chain Reaction
PIVKA-II	Protein Induced by the Absence of Vitamin K Factor II

**(S)AE**	**(Serious) Adverse Event**
**SMC**	**Safety Monitoring Committee**
**SPC**	**Summary of Product Characteristics; in Dutch: officiële productinformatie IB1-tekst**
**Sponsor**	**The sponsor is the party that commissions the organisation or performance of the research, for example a pharmaceutical company, academic hospital, scientific organisation or investigator. A party that provides funding for a study but does not commission it is not regarded as the sponsor, but referred to as a subsidising party.**
**TNF**	**Tumor Necrosis Factor**
**UAVG**	**Dutch Act on Implementation of the General Data Protection Regulation; in Dutch: Uitvoeringswet AVG**
**VKORC1**	**Vitamin K epoxide reductase complex subunit 1**
**VKA**	**Vitamin K antagonist**
**WMO**	**Medical Research Involving Human Subjects Act; in Dutch: Wet Medisch-wetenschappelijk Onderzoek met Mensen**

SUMMARY

Rationale: Coronavirus disease 2019 (COVID-19) is caused by the severe acute respiratory syndrome coronavirus-2 (SARS-CoV-2). While the majority of people recover after mild symptoms, a portion of COVID-19 patients develops respiratory failure. Coagulopathy and thromboembolism are prevalent in severe COVID-19, and these factors are associated with decreased survival. Coagulation is an intricate balance between clot promoting and dissolving processes in which vitamin K plays an essential role. Elastin is a major component of dynamic tissues such as lungs and arteries, and elastin calcification stimulates elastin degradation and *vice versa*. The vitamin K-dependent Matrix Gla Protein (MGP) protects elastin from both calcification and degradation.

Although technically feasible, direct quantification of blood vitamin K levels is not an appropriate method to assess overall vitamin K status due to differences in bioavailability and half-life time between the two naturally occurring vitamin K forms (vitamin K1 and K2). Measuring inactive levels of vitamin K-dependent proteins in the circulation is the method recommended by most experts, as it represents the systemic availability of both vitamin K1 and K2. Dp-uc (dephospho uncarboxylated, i.e. inactive) MGP and proteins induced by vitamin K absence (PIVKA-II) both inversely correlate with vitamin K status and can be used as surrogate markers of total vitamin K status.

Recently, we found a severely reduced vitamin K status (as quantified by dp-ucMGP) in COVID-19 patients compared to controls. In COVID-19 patients, low vitamin K status was also associated with poor outcome (defined as the need for invasive ventilation or death), accelerated elastin degradation (quantified by plasma (iso)desmosine (DES) a byproduct of elastin degradation). Based on these finding and previous studies, we hypothesize that improving vitamin K-status by vitamin K supplementation could have favorable effects on pulmonary damage and coagulopathy in COVID-19.

Objectives

Primary objectives:

- To evaluate the effect of oral vitamin K2 supplementation on elastic fiber degradation, as measured by plasma desmosine, during COVID-19 requiring hospital admission
- To evaluate the effect of oral vitamin K2 supplementation on extrahepatic vitamin K status, as measured by circulating dp-ucMGP, during COVID-19 requiring hospital admission

Secondary objectives:

- To evaluate the effects of oral vitamin K2 supplementation on adverse events and disease progression during COVID-19 requiring hospital admission
- To evaluate the effects of oral vitamin K2 supplementation on hepatic vitamin K status, as measured by circulating PIVKA-II, during COVID-19 requiring hospital admission

Exploratory objectives:
- To evaluate the effect of vitamin K2 supplementation on circulating (undercarboxylated) protein S
- To evaluate the effect of oral vitamin K2 supplementation on respiratory failure requiring intubation and mechanical ventilation or leading to death
- To evaluate the effects of supplementation of oral vitamin K2 on disease severity on lung CT scan
- To evaluate the effect of oral vitamin K2 supplementation on active MGP concentrations
- To evaluate the effect of oral vitamin K2 supplementation on TFPI and normative APC sensitivity ratio and prothrombin (Echis Carinato test)
- To evaluate the effect of oral vitamin K2 on circulating MMP9
- To evaluate the effect of oral vitamin K2 on systemic inflammation by measuring cytokines and inflammatory mediators like interleukin-6 (IL-6)
- To further explore the pathophysiology and biomarkers for COVID-19 susceptibility, severity and disease progression, including Vitamin K epoxide reductase complex subunit 1 (VKORC-1) gene polymorphism

Study design: This is a phase 2a, double-blind randomized placebo-controlled intervention trial (1:1 randomization).

Study population: A total of 40 patients with respiratory failure due to (proven) COVID-19 disease who are admitted to the Canisius Wilhelmina Ziekenhuis (CWZ) in Nijmegen, the Netherlands will be enrolled.

Inclusion criteria
- COVID-19 patients who are admitted to the CWZ with COVID-19 as primary reason for admission, with a laboratory confirmed SARS-CoV-2 infection within the previous 14 days

- Respiratory failure requiring supplemental oxygen, defined as requiring supplemental oxygen to sustain an arterial PO2 ≥70mmHg (measured by arterial blood gas) or an oxygen saturation of ≥94% (measured using a pulse oximeter)
- At least 18 years old
- Able to safely swallow the study medication or possibility of safely administering this through a nasogastric tube
- Use of prophylactic heparin or LWMH according to hospital protocols, or use of therapeutic dosages if there is a medical indication for this
- Informed consent signed by patient

Exclusion criteria

- Use of oral anticoagulation drugs; patients may be included when they have been switched to LMWH
- Patients on vitamin K antagonists with a supra-therapeutic anticoagulation at admission who require vitamin K supplementation to correct this, or were administered vitamin K for this reason within the preceding 5 days
- Patients already using vitamin K supplements at admission
- Participation in another intervention study
- Direct admission to an intensive care unit (ICU) for invasive ventilation at presentation.
- Confirmed active pulmonary embolism or deep venous thrombosis prior to inclusion
- Known allergy to any of the components of the study medication or placebo
- Patients who are hemodialysis dependent at admission
- Pregnancy at the time of inclusion
- Diagnosed active malignancy at the time of inclusion

Intervention

Patients will take three tablets of either vitamin K2 menaquinone-7 (333mcg) or placebo per day. Patients taking vitamin K2 MK-7 will receive the total of 999mcg per day from day 1 until day 14 or discharge, whichever occurs earlier. All subjects can be treated with prophylactic or therapeutic heparin-based (heparin or any low-molecular weight heparin) anticoagulants, according to local hospital protocols.

Main study parameters/endpoints

<u>Primary endpoints:</u>

- Plasma desmosine levels before and during vitamin K supplementation in intervention versus control patients

- Plasma dp-uc MGP levels before and during vitamin K supplementation within the intervention group and in intervention versus control patients

Secondary endpoints

- Difference between the number of grade 3 and grade 4 adverse events between the intervention and control group during treatment, with special attention for: progression of respiratory insufficiency, thrombotic events, pulmonary embolism or deep venous thrombosis, bleeding, renal insufficiency, cardiac decompensation, liver enzyme abdnormalities and/or liver failure
- Serum PIVKA-II levels before and during vitamin K supplementation in intervention versus control patients

Exploratory endpoints:

- Serum (undercarboxylated) protein S levels before and after vitamin K supplementation
- Incidence of respiratory failure defined as either intubation and mechanical ventilation or death (with respiratory failure as a likely direct or indirect cause of death) in the intervention versus control group
- Change in disease severity as measured by low-dose chest CT on day 5 and 10 versus baseline in intervention versus control groups
- Plasma levels of active MGP before and after vitamin K supplementation
- Normative APC sensitivity ratio and prothrombin (Echis Carinato test) and TFPI concentration before and after vitamin K supplementation
- Inflammatory parameters including CRP, d-dimer, ferritine, IL-6, TNF-α, IFN-γ and s-IL-2r as measured by ELISA or Multiplex in both intervention and control groups
- Other markers related to the pathogenesis, susceptibility and severity of COVID-19 in both intervention and control groups
- Vitamin K epoxide reductase complex subunit 1 (VKORC-1) gene polymorphisms and vitamin D receptor gene polymorphisms

Follow-up: Clinical parameters, including symptoms, vital signs, grade 3 or 4 adverse events and serious adverse events will be collected daily during admission. Mortality will be determined 28 days after inclusion.

At baseline (up to 24 hours before treatment initiation) and subsequently three times a week (i.e. day 1, 3, 5 etc.) the following laboratory tests are performed immediately: full blood count, electrolytes (sodium, potassium, calcium), creatinine, c-reactive protein (CRP), and albumin. At each timepoint EDTA and citrate plasma, and serum will be frozen and stored at -80 degrees Celsius. At each time point (iso)desmosine (DES, a marker for elastin degradation, in EDTA plasma), dp-ucMGP (inversely associated with vitamin K status, in

EDTA plasma), PIVKA-II (inversely associated with vitamin K status, in serum) will be determined.

Further exploratory measurements can be performed on these, or a subset of these samples, including: (undercarboxylated)-protein S (measured by ELISA in citrate plasma), active MGP (measured by ELISA), protrombin (Echis Carinatus test), TFPI (measured by ELISA in citrate plasma), normalized APC sensitivity ratio (nAPCsr) (protein S and TFPI activity), 25-OH vitamin D, plasma cytokines (i.e. IL-6, TNF-α, IFN-γ), soluble IL-2-receptor (s-IL-2r) and antibodies to SARS-CoV-2.

Computed tomography (CT) is performed at baseline and on day 5 after study start for assessing pulmonary involvement score using published protocols. If patients are discharged before day 5 a second CT scan is made prior to discharge. Severity of Covid-19 pneumonia will be quantified and expressed as volume percentage of lung involvement on low dose chest CT by a specialized chest radiologist at University Medical Center Utrecht (UMCU).

At one point during the trial patient DNA will be collected using a mouth swab, for determination of the VKORC1 gene polymorphism.

Nature and extent of the burden and risks associated with participation, benefit and group relatedness:

Benefits: There is a potential benefit for participants in this study if vitamin K2 is shown to help prevent complications from COVID-19 disease. However, there is no preliminary causal evidence for this relationship. Participants will be randomized 1:1 in the study and intervention group, meaning only half will receive the intervention.

Risks and burdens: Risks for participants are related to three interventions: 1) taking vitamin K2, 2) repeated blood draws, 3) extra low-densitiy CT scan(s). To date no adverse side-effects from oral vitamin K2 supplementation have been described in persons who do not use vitamin K antagonists (VKAs). However, there is no data on the use of vitamin K during COVID-19 disease. Blood will be drawn by venipuncture or through an intravenous cannula at regular time points as part of routine patient care. In study participants 25ml extra blood will be drawn every two days during admission. This volume is not expected to have consequences for the health of the patients. Finally, in participants one or two low dose CTs will be made outside routine care for COVID-19. The risks of this are expected to be minimal as the radiation exposure of a single low-dose CT is 1mSv.

1. INTRODUCTION AND RATIONALE

1.1 Background

COVID-19

Coronavirus 2019 disease (COVID-19) is an infectious disease caused by the severe acute respiratory syndrome (SARS) coronavirus (CoV)-2. The majority of individuals who contract SARS-CoV-2 have only mild symptoms, but a significant proportion develops respiratory failure due to severe pneumonia and/or acute respiratory distress syndrome (ARDS) [1]. COVID-19 may also have extrapulmonary manifestations, including coagulopathy and venous thromboembolism, associated with decreased survival [2]. The pathogenesis of this coagulopathy, and the links between pulmonary and thromboembolic manifestations of COVID-19 are incompletely understood.

Vitamin K in coagulation and elastic fiber metabolism

Coagulation is an intricate balance between clot promoting and dissolving processes in which vitamin K plays a well-known role. By far the majority of research on vitamin K has focused on its role in activating hepatically synthesized pro-coagulation factors II (i.e. thrombin), VII, IX and X. These proteins depend on vitamin K for carboxylation to fulfill their biological function. As such antagonizing the activity of vitamin K, using so-called vitamin K antagonists (VKAs), such as acenocoumarol, results in decreased coagulation.

However vitamin K is also a cofactor of anticoagulant protein C and protein S. In contrast to the pro-coagulant factors and protein C, a significant proportion of protein S is extrahepatically synthesized in endothelial cells. This endothelial protein S plays a local suppressive role against thrombosis [3].

Matrix Gla protein (MGP) is also vitamin K-dependent but not involved in coagulation [4]. MGP has been extensively studied as an inhibitor of vascular mineralisation [5], and its role in the pulmonary compartment seems to be comparable [6, 7]. Besides preventing soft tissue calcification, MGP also protects against elastic fiber degradation. Elastic fibers are essential matrix components in lungs and have high calcium affinity [8]. Degradation and mineralization of elastic fibers are interrelated processes [9, 10].

Storage capacity of vitamin K is limited, and therefore its metabolism must be very efficient. After being oxidized during the carboxylation reaction, vitamin K is reactivated repeatedly by the enzyme vitamin K epoxide reductase (VKOR) in the vitamin K cycle [11]. Nevertheless, insufficiency may develop within days of poor intake, particularly in pathological states of increased vitamin K utilization [7, 12, 13].

Triage theory regarding vitamin K

During times of scarcity, micronutrients are reserved for use in processes that form the greatest threat to short-term survival if not properly executed [14]. With regard to vitamin K insufficiency, it appears to be preferentially transported to the liver for the activation (via carboxylation) of procoagulant factors at the expense of extrahepatic vitamin K-dependent proteins such as MGP and protein S (figure 1) [15].

This was demonstrated in women between 60 and 80 years old who consumed a vitamin K1 deficient diet for 28 days. Undercarboxylated osteocalcin, a vitamin K-dependent bone protein, increased almost immediately, whereas undercarboxylated factor II increased more slowly [15]. Moreover, when patients using vitamin K antagonists (VKA) as anticoagulants steadily increased their dietary intake of vitamin K1, a significant decrease in undercarboxylated factor II was seen at 150 µg/day, while a significant decrease in undercarboxylated osteocalcin was only seen at an intake 300 µg/day [16].

Similar to osteocalcin and MGP, vitamin K insufficiency would result in deficient activation of endothelial protein S before causing a decrease in carboxylated prothrombin [17]. This could explain the seemingly paradoxical increase of thrombosis risk in the first week of treatment with VKAs [18].

Assessment of vitamin K status

Measuring circulating levels of the two naturally occurring forms of vitamin K – vitamin K1 (phylloquinone) and K2 (the group of menaquinones) – is technically feasible. However, the value of such measurements is limited. Quantification of vitamin K-dependent proteins that have not been carboxylated, on the other hand, is a valuable method reflecting the combined functional deficit of vitamin K1 and K2. Determination of desphospho-uncarboxylated (dp-uc; i.e. inactive) MGP levels as well as the ratio between uncarboxylated and carboxylated osteocalcin are validated assays of extrahepatic vitamin K status [19].

Dp-ucMGP is a robust biomarker of extrahepatic vitamin K status. High dp-ucMGP reflects low vitamin K status and vice versa. Although increasing vitamin K consumption decreases dp-ucMGP [20-22], its levels are not simply a reflection of vitamin K intake but depend on other factors as well. Circulating dp-ucMGP concentration can best be regarded as a reflection of the total extrahepatic vitamin K deficit, i.e. the amount of vitamin K that is needed to carboxylate all the uncarboxylated vitamin K-dependent proteins in the body [23].

Hepatic vitamin K status is usually quantified by measuring levels of protein induced by vitamin K absence (PIVKA)-II (i.e. uncarboxylated prothrombin) [15].

1.2 Rationale for the current study

Recently, we found a reduced vitamin K status (as quantified by dp-ucMGP) in COVID-19 patients as compared to controls. In these patients, low vitamin K status was also associated with poor outcome (defined as the need for invasive ventilation or death) and accelerated elastic fiber degradation (quantified by plasma (iso)desmosine (DES) a byproduct of elastin degradation). In contrast, hepatic vitamin K status, measured by inactive factor II (also called protein-induced by vitamin K absence (PIVKA)-II) was unaffected in most patients (figure 2).

Low dietary vitamin K intake and VKA use are evident causes of elevated dp-ucMGP in the general population [13, 24]. However, during COVID-19, inflammation causes pulmonary elastic fiber damage which could also lead to an upregulation of MGP and a draining of extrahepatic vitamin K. The significant correlation between increased dp-ucMGP levels, and elastic fiber degeneration and poor prognosis supports the theory that vitamin K insufficiency contributes to pulmonary pathology.

Though dp-ucMGP is elevated, hepatic pro-coagulant vitamin K status, quantified by measuring PIVKA-II, was hardly affected. Considering the preferential activation of hepatic- over extrahepatic proteins and the fact that about 50% of protein S synthesis and activation by vitamin K occurs in endothelial cells, this could increase the risk of coagulation. Consumption of clotting factors during thrombosis would put a further burden on vitamin K stores by increasing demand for activation of newly synthesised coagulation factors. With preference given to the carboxylation of pro-coagulant factors, progressive depletion of active endothelial protein S increasingly skews the balance towards coagulation.

Based on these finding and previous studies, we hypothesize that improving vitamin K-status by vitamin K supplementation could have favorable effects on both pulmonary damage and coagulation abnormalities in COVID-19 patients.

There is increasing evidence that vitamin K2 differs from vitamin K1 in terms of absorption, half-life and carboxylation efficiency, recently reviewed in [25]. In short vitamin K2, especially in the form of menaquinone-7 (MK-7), has a longer half-life and more potent activity in the carboxylation of vitamin K-dependent proteins. As a consequence most studies investigating the health benefit of vitamin K on outcomes for osteoporosis, atherosclerosis, cancer and inflammatory diseases have used vitamin K2 MK-7 [25].

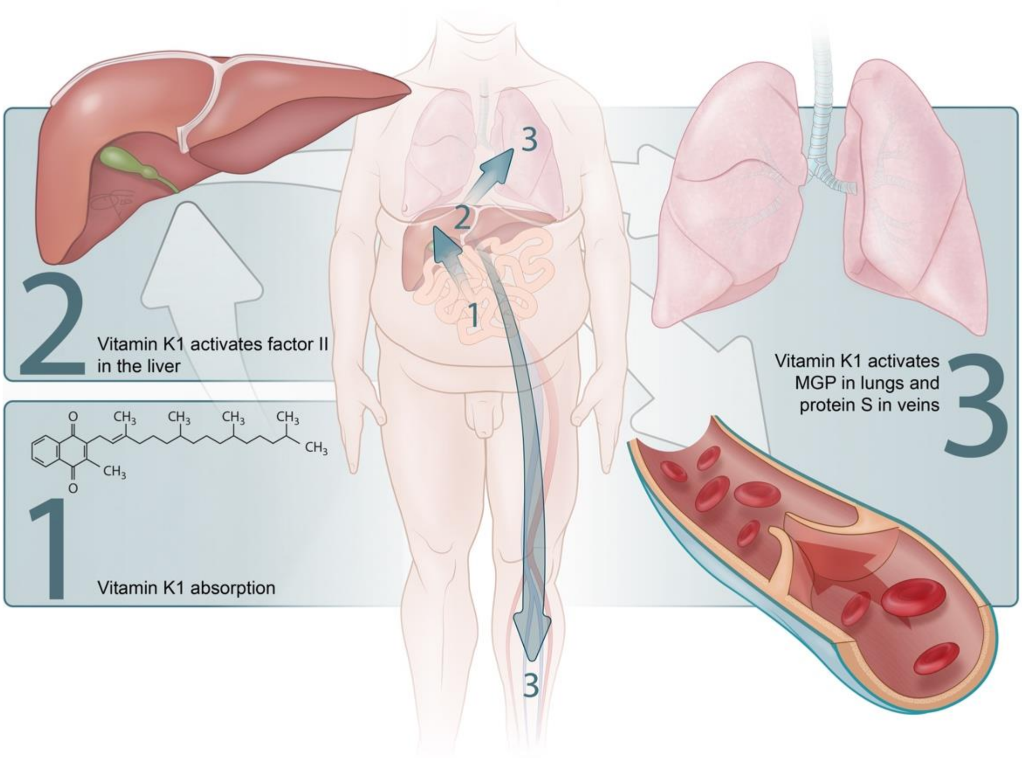

Figure 1: (1) After absorption, vitamin K1 is preferentially transported to the liver via the portal circulation, where it is utilized for carboxylation of hepatic coagulation factors. This implies that during periods of vitamin K insufficiency, (2) the grade of carboxylation is usually higher for hepatic factor II (3) than for endothelial protein S in veins and pulmonary matrix Gla protein (MGP). Source: [26].

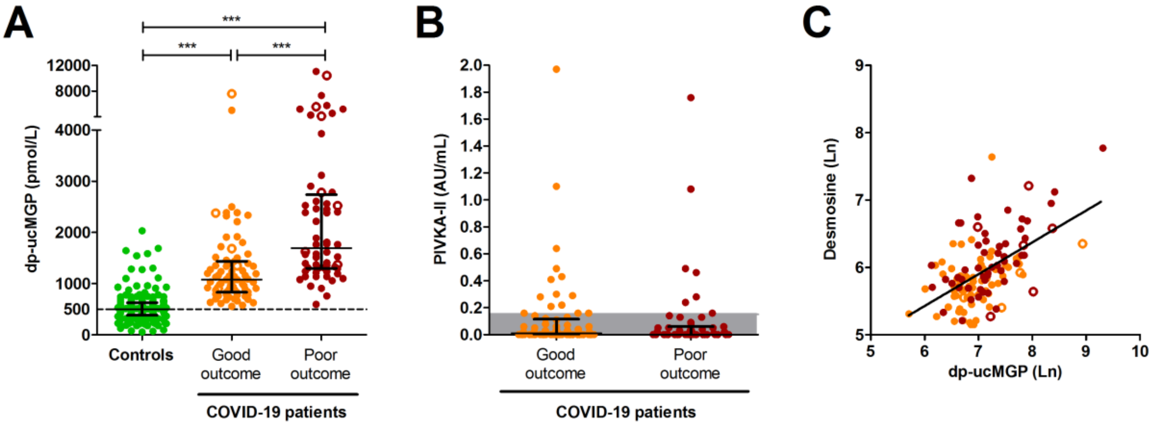

Figure 2: **(A)** Dp-ucMGP was measured in plasma of COVID-19 patients with a good outcome (discharge without invasive ventilation, n=75, orange) or poor outcome (invasive ventilation and/or death, n=60, red), compared to a cohort of controls. Open circles represent patients using VKA at admission. **(B)** PIVKA-II was measured in those patients not using

VKA (n=122). The detection threshold and normal range for healthy controls is shown in gray. **(C)** For all COVID-19 patients who were not dialysis dependent at admission with a good outcome (n=69, orange) or poor outcome (n=58, red) log-transformed baseline dp-ucMGP and desmosine values are shown, with open circles representing VKA users. Figures modified from [26].

2. OBJECTIVES

Primary objectives:

- To evaluate the effect of oral vitamin K2 supplementation on elastic fiber degradation, as measured by plasma desmosine, during COVID-19 requiring hospital admission
- To evaluate the effect of oral vitamin K2 supplementation on extrahepatic vitamin K status, as measured by circulating dp-ucMGP, during COVID-19 requiring hospital admission

Secondary objectives:

- To evaluate the effects of oral vitamin K2 supplementation on adverse events and disease progression during COVID-19 requiring hospital admission
- To evaluate the effects of oral vitamin K2 supplementation on hepatic vitamin K status, as measured by circulating PIVKA-II, during COVID-19 requiring hospital admission

Exploratory objectives

- To evaluate the effect of vitamin K2 supplementation on circulating (undercarboxylated) protein S
- To evaluate the effect of vitamin K2 supplementation on respiratory failure requiring intubation and mechanical ventilation or leading to death
- To evaluate the effects of supplementation of vitamin K2 on disease severity on lung CT scan
- To evaluate the effect of vitamin K2 supplementation on active MGP concentrations
- To evaluate the effect of vitamin K2 supplementation on TFPI and normative APC sensitivity ratio and prothrombin (Echis Carinato test)
- To evaluate the effect of vitamin K2 on circulating MMP9
- To evaluate the effect of vitamin K2 on systemic inflammation by measuring cytokines and inflammatory mediators like interleukin-6 (IL6)
- To further explore the pathophysiology and biomarkers for COVID-19 susceptibility, severity and disease progression, including the Vitamin K epoxide reductase complex subunit 1 (VKORC-1) gene polymorphism and vitamin D receptor gene polymorphism

3. STUDY DESIGN

This is a double-blind, placebo-controlled randomized phase 2a intervention study in male and female patients over 18 years old with COVID-19 requiring supplemental oxygen and hospital admission. Patients will be treated with oral vitamin K2 or placebo from day 1 until discharge, with an expected average hospital stay duration of 6 days. After written informed consent is obtained from the patients or a legal representative, eligible patients will be randomized between the intervention (vitamin K2, in the form of menioquine-7) and control (placebo) groups.

Patients randomized to the intervention group will take three tablets of vitamin K2 menaquinone-7 333mcg per day (for a total dose of 999mcg per day), from day 1 until day 14 or discharge, whichever occurs first, figure 3. Patients in the control group will receive a matched placebo. Adverse events will be collected daily and blood will be sampled three times a week until discontinuation of treatment. A low-dose chest CT scan will be made at admission and 5 days after treatment. If a patient is discharged before day 5 post treatment, a CT scan will be made before discharge. Mortality and serious adverse events will be followed up on day 28.

Patients will receive all routine treatment for COVID-19 according to general hospital practice.

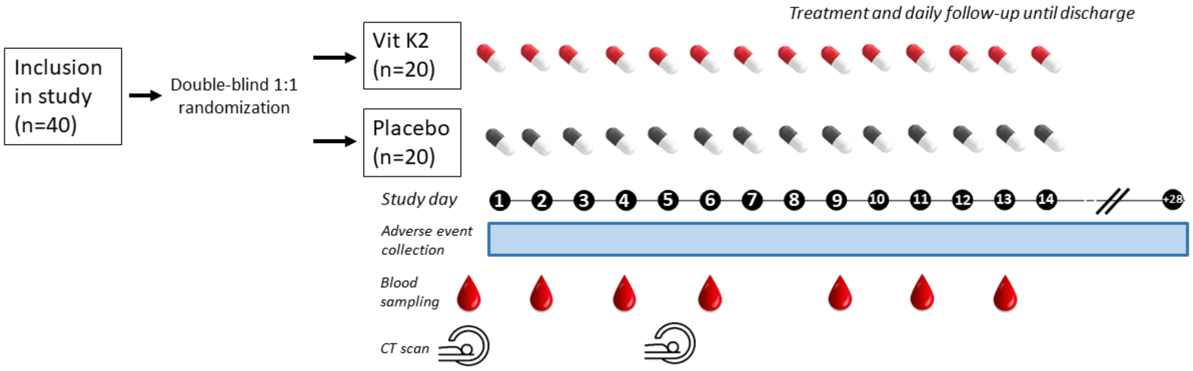

Figure 3: Clinical trial design.

4. STUDY POPULATION

4.1 Population (base)

This study will enroll 40 male and female patients aged 18 years or older, who are admitted to the hospital with PCR-proven COVID-19 requiring supplemental oxygen.

4.2 Inclusion criteria

In order to be eligible to participate in this study, a subject must meet all of the following criteria:

- COVID-19 patients who are admitted to the CWZ with COVID-19 as primary reason for admission, with a laboratory confirmed SARS-CoV-2 infection within the previous 14 days
- Respiratory failure requiring supplemental oxygen, defined as requiring supplemental oxygen to sustain an arterial PO2 ≥70mmHg (measured by arterial blood gas) or an oxygen saturation of ≥94% (measured using a pulse oximeter)
- At least 18 years old
- Able to safely swallow the study medication or possibility of safely administering this through a nasogastric tube
- Use of prophylactic heparin or LWMH according to hospital protocols, or use of therapeutic dosages if there is a medical indication for this
- Informed consent signed by patient

4.3 Exclusion criteria

A potential subject who meets any of the following criteria will be excluded from participation in this study:

- Use of oral anticoagulation drugs; patients may be included when they have been switched to LMWH
- Patients on vitamin K antagonists with a supra-therapeutic anticoagulation at admission who require vitamin K supplementation to correct this, or were administered vitamin K for this reason within the preceding 5 days
- Patients already using vitamin K supplements at admission
- Participation in another intervention study
- Direct admission to an intensive care unit (ICU) for invasive ventilation at presentation
- Confirmed active pulmonary embolism or deep venous thrombosis prior to inclusion
- Known allergy to any of the components of the study medication or placebo
- Patients who are hemodialysis dependent at admission
- Pregnancy at the time of inclusion

- Diagnosed malignancy at the time of inclusion

4.4 Sample size calculation

This is the first study evaluating the effects of oral vitamin K in COVID-19 patients. In accordance with standards for early phase clinical trials the primary outcomes of this study were chosen to facilitate a study with a relatively small sample size.

The key primary outcome will be the effect of vitamin K supplementation on elastic fiber degradation, determined by measuring plasma desmosine (pDES) in the intervention versus the control group. In the previous study of 135 COVID-19 patients pDES has a log-normal distribution, with a mean of ln of 5.99 with a standard deviation of 0.603. The effects of vitamin K2 on this outcome have never been studied, however, we consider a difference in ln(pDES) of 10% to be clinically significant. An independent samples t-test (with $\alpha=0.05$ and $\beta=0.20$) would be statistically significant between two groups of 16 persons (total study population 32 persons).

Based on these two outcomes the sample size of 40 persons allows for a 10% loss to follow-up for the per-protocol analysis.

5. TREATMENT OF SUBJECTS

5.1 Investigational product

Patients will take three tablets of either vitamin K2 menaquinone-7 (333mcg) or placebo per day. Patients taking vitamin K2 MK-7 will receive the total of 999mcg per day from day 1 until day 14 or discharge, whichever occurs earlier.

While concomitant use of oral anticoagulant drugs is an exclusion criteria, all subjects will be treated with prophylactic heparin-based (heparin or any low-molecular weight heparin) anticoagulants according to local hospital protocols.

5.2 Escape medication

If patients develop thromboembolic complications or have another indication for anticoagulant treatment (such as atrial fibrillation) they can be treated with therapeutic dosages of heparin-based (heparin or any low-molecular weight heparin) anticoagulants without being excluded from the study.

6. INVESTIGATIONAL PRODUCT

6.1 Name and description of investigational product(s)

Investigational product: Vitamin K2, in the form of menaquinone-7 (MK-7), 'Vitamin K2 MK-7' or placebo containing only inactive ingredients.

Structure and function

Vitamin K represents a family of fat-soluble vitamins with the common chemical structure of 3-substituted 2-methyl-1,4-napthoquinone. It is found naturally in food as phylloquinone (vitamin K1) and menaquinones (vitamin K2). Phylloquinone is the primary form of vitamin K found in typical Dutch diets: it is found in dark green leafy vegetables (e.g. spinach, lettuce andother salad plants). Menaquinones are a group of compounds with an unsaturated sidechain from 4 to 13 isoprenyl units (vitamin K2 or MK-n). They are found mainly in animal productssuch as meat, cheese and eggs, and in a fermented soybeans called 'natto'. The vitamin K2 series is synthesized by various Gram-positive bacteria present in the jejunum and ileum [27].

Vitamin K acts as a cofactor for the reaction in which the enzyme γ-glutamyl carboxylase (GGCX) catalyses the carboxylation of glutamic acid (Glu) residues into γ-carboxyglutamic acid (Gla) residues in vitamin K-dependent proteins(Gla-proteins), which is required for their activity. These Gla-proteins are involved in many physiological processes, including blood coagulation and anticoagulation, elastic fiber metabolism and bone mineralisation. MK-7 may have a greater bioactivity than phylloquinone and other menaquinones [27].

Absorption, bioavailability and metabolism

Vitamin K is readily absorbed. Absorption of vitamin K takes place in the proximal small intestine by a saturable, energy dependent process. Absorption of vitamin K is enhanced by bile salts and pancreatic juice. Overall, 40-70% of vitamin K may be absorbed in the jejunum.

Vitamin K is transported via the lymph in chylomicrons and concentrated in the liver prior to wide distribution. The body pool of vitamin K is low. The metabolism of vitamins K1 involves the formation of an epoxide and a quinone, which can then be reduced by NAD(P)H reductases. The reduced form is involved in carboxylation reactions. The carboxylic acid derivatives of vitamin K1 are conjugated with glucuronic acid. In rats, vitamin K1 can be converted to K2 in the tissues.

Approximately 30-40% of vitamin K is excreted, via the bile, in the faeces as partially degraded, conjugated water-soluble metabolites. Smaller quantities (approximately 15%) are excreted in the urine as water-soluble metabolites.

The pharmacodynamic and pharmakinetic properties of the vitamin K forms differ, figure 4. Vitamin K2 MK-7 has a greater activity and a longer half-life than vitamin K1 or MK-4 [25].

Vitamin K isoform	Structure	$K_{1/2}$ in cells	$T_{1/2}$ in blood	Primary source in food
**Vitamin K1** Phylloquinone (K-1)	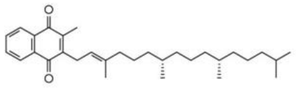	12.3μM	~ 3 hours	Leafy greens e.g. spinach, kale
**Vitamin K2** Menaquinone-4 (MK-4)	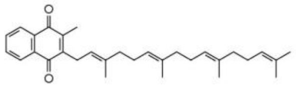	3.6μM	~ 1.5 hours	Meat and dairy e.g. beef, poultry, butter, soft cheese
**Vitamin K2** Menaquinone-7 (MK-7)	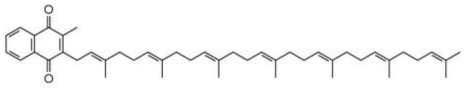	1.9μM	~ 70 hours	Fermented foods and dairy e.g. natto, hard cheese, sauerkraut
**Vitamin K2** Menaquinone-8 (MK-8)	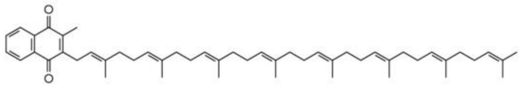	1.4μM	~ 70 hours	Fermented foods and dairy e.g. natto, hard cheese, sauerkraut

Figure 4: Molecular structures, $K_{1/2}$ and $T_{1/2}$ of different vitamin K forms. Source: [25].

Reference values and toxicity

In a recent publication the European Food Safety Authority noted that there was insufficient data to provide guidance on Daily Reference Values for vitamin K intake. However, they also note that there are no studies that show adverse effects associated with supplementation [27].

Investigational product

Kappa Biosciences AS (Norway) manufactures a synthetic form of Vitamin K2 MK-7 (a.k.a.: K2VITAL®). Vitamin K2 MK-7 is manufactured by Kappa Biosciences AS in Norway as a pure crystalline powder. Since the amount of the active ingredient per tablet is so low, it is shipped to BASF AS (Denmark) for preparation of a formulation suitable for manufacturing of tablets. This is a microencapsulated Vitamin K2 MK-7 product with inactive ingredients (see documents in Section D). The active, microencapsulated product is further shipped to Optipharma (Norway) who manufacture tablets containing 333 μg Vitamin K2 MK-7 and placebo tablets. Finished tablets will be packed by Optipharma and labelled by Kappa Bioscience AS.

Vitamin K2 is registered for use as ingredient in food supplements in the Netherlands and has also been approved by the European Food Safety Authority (EFSA). The manufacturer will assess the uniformity and stability of the finished product.

6.2 Summary of findings from non-clinical studies

The only well-described function of vitamin K is to serve as a cofactor for the enzyme γ-glutamate carboxylase (GGCX) during the posttranslational carboxylation of glutamate residues into γ-carboxyglutamate (Gla) [28]. Proteins undergoing this carboxylation are known as Gla-proteins. Currently 17 Gla-proteins have been identified, and in all cases in which their function is known, the carboxylation step is a prerequisite for their biological activity [29].

After its initial discovery in liver, GGCX was found to be present in almost all mammalian tissues including lung, heart, kidney, spleen, testis, cartilage and bone [30]. It was also identified in skin, both in the dermis and in the epidermis [31]. Most Gla-proteins are expressed in only one type of tissue, such as the procoagulant factors II, VII, IX and X which are all synthesized in the liver, and osteocalcin, which is uniquely synthesized in bone [32]. MGP, on the other hand, has a much wider expression pattern and was identified in most extrahepatic tissues in which also GGCX was found [6]. Different functions have been reported for the various Gla-proteins: the most well-known are those involved in hemostasis, such as prothrombin and the clotting factors VII, IX and X [32].

Another family of Gla-proteins is that of the calcification inhibitors: osteocalcin, MGP and Gla-rich Protein (GRP, also known as UCMA) [33]. MGP is a strong inhibitor of soft tissue calcification and was also found to be associated with the elastic fibers, for instance in the dermis [34] and in the large arteries [4], where it is synthesized by fibroblasts [35] and vascular smooth muscle cells [36], respectively. As was demonstrated by Price, small calcification inhibitors like osteocalcin and MGP are essential in the prevention of elastin and collagen fibril calcification, a principle that was designated as mineralization by inhibitor exclusion [37]. Poor vitamin K status has been demonstrated to be associated with vascular stiffening [38] and excessive calcium salt precipitation, both in animal models [39] and in humans [40]. It is to be expected that a similar mechanism is operational in the elastin fibrils in the lung.

Toxicity studies were conducted in mice, chicks and rats. Vitamin K1 in single oral doses up to 25,000 mg/kg BW, produced no fatalities in mice. Daily doses as high as 2000 mg/kg body weight vitamin K1 produced no ill effects [41]. In an acute oral toxicity test, mice were given a single oral dose of 2000 mg/kg body weight Vitamin K2 MK-7 (limit dose) where no toxicities were observed during the 14-day observation period. In a subchronic oral toxicity test in rats, animals were administered Vitamin K2 MK-7 for 90 days by gavage at the following doses: 0 (vehicle control, corn oil), 2.5, 5, and 10 mg/kg body weight per day. The no observed adverse effect level (NOAEL) of MK-7, when administered orally to rats for 90 days, was considered to be equal to 10 mg/kg body weight per day, the highest dose tested, based on lack of toxicity [42, 43].

6.3 Summary of findings from clinical studies

A Japanese dish called ‘natto’ that consists of fermented soy beans and is extremely rich in vitamin K2 has been eaten for nearly 1000 years. It has been postulated that this may be the reason for the low incidence of osteoporosis in these areas [25]. Several studies have been conducted in healthy volunteers consuming natto daily. The amount of natto consumed by participants varies between studies often contained up to 900µg vitamin K2 per day without any adverse effects [44-47]. These studies demonstrated a rapid increase in serum MK-7 after ingestion, figure 5 [48].

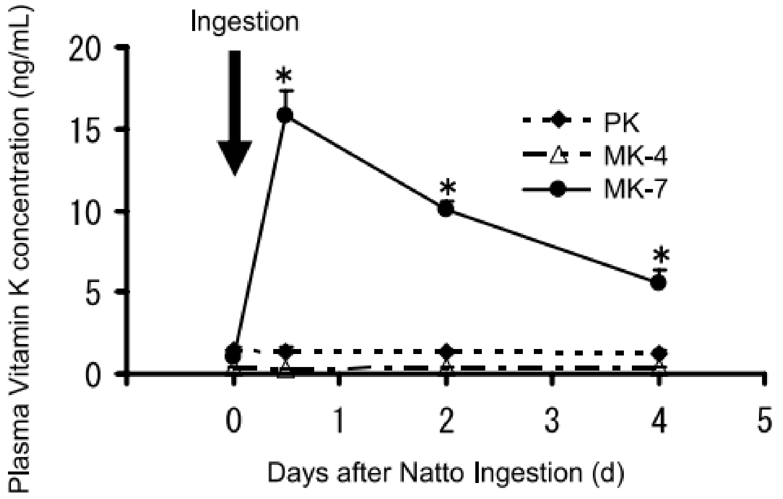

Figure 5: Plasma concentrations of vitamin isoforms (phylloquinone, menaquinone-4 and menaquinone-7) in n=5 subjects before and up to 4 days after eating one package of natto (50 grams) [48].

Furthermore, in studies where the ingested natto had an equivalent of around 900µg Vitamin K2 MK-7 per day a rapid increase both in plasma levels of MK-7 and an increase in carboxylated osteocalcin (a vitamin K dependent bone protein) were seen as early as 1 day after ingestion, figure 6 panel A and B [47]. This effect already appears to be near its maximum on day 7 after starting, with only a small not statistically significant increase, between day 7 and day 14, figure 6 panel C and D [46].

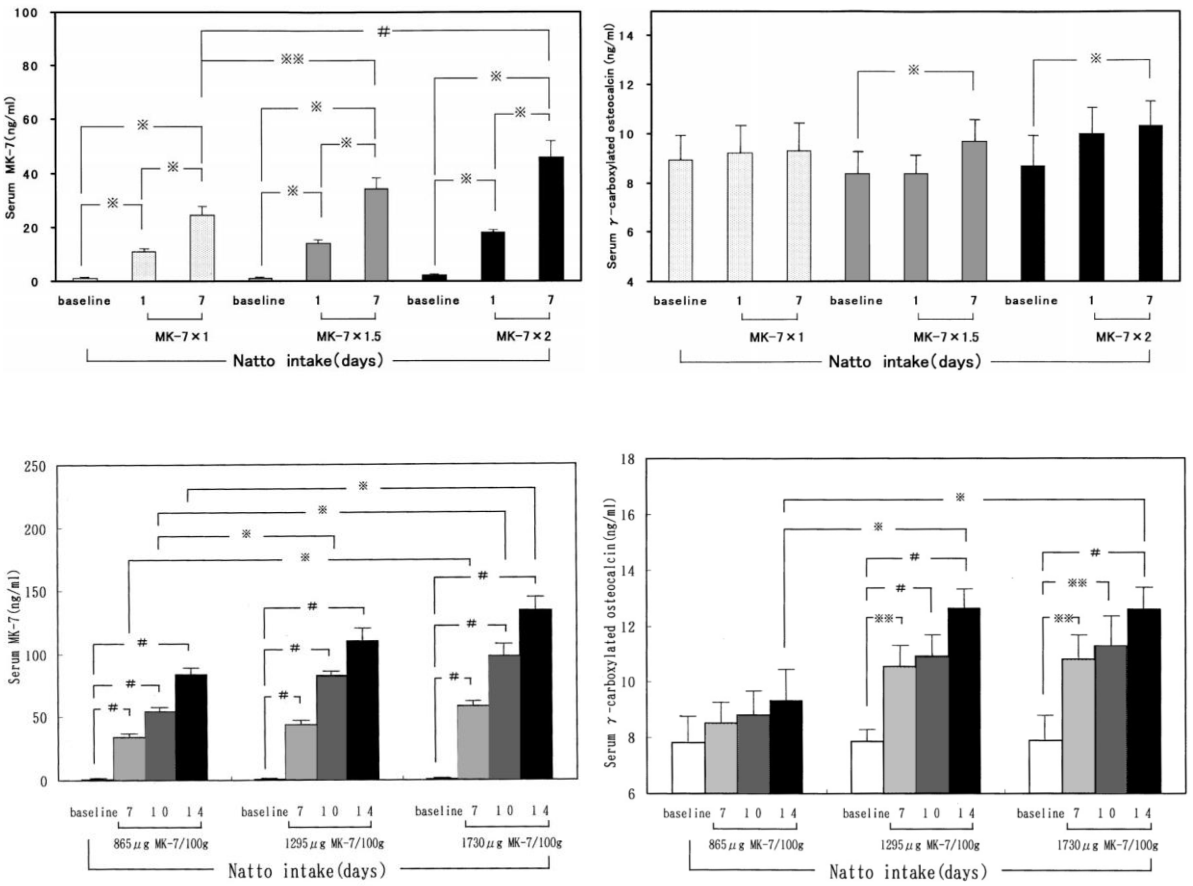

Figure 6: Panel A and B: N=8 healthy adults ingested 50 grams of natto containing 775µg/100g, 1289µg/100g or 1765µg/100g vitamin K2 MK-7 once a day for 7 days. Serum MK-7 and γ-carboxylated osteocalcin (osteocalcin activated by vitamin K) were measured at baseline and after 1 and 7 days [46]. Panel C and D: N=16 healthy adults ingested 50 grams of natto containing 865µg/100g, 1295µg/100g or 1765µg/100g vitamin K2 MK-7 once a day for 14 days. Serum MK-7 and γ-carboxylated osteocalcin (osteocalcin activated by vitamin K) were measured at baseline and after 7, 10 and 14 days [47].

Many clinical trials have been conducted using vitamin K2, recently reviewed in [25]. These studies consistently show that vitamin K2 administration reduces the levels of uncarboxylated vitamin-K-dependent proteins, including dp-ucMGP, PIVKA-II and ucOC.

A number of trials, and available systematic reviews, have demonstrated significant effect of vitamin K2 in reducing osteoporotic fractures in postmenopausal women. Despite dosages as high as 45mg vitamin K2 (in the form of menaquinone-4) per day, these trials have not reported adverse events, including thrombotic or bleeding events [49].

Vitamin K deficiency has been well established in hemodialysis patients and has been implicated as the link between fractures and vascular calcification in these populations. As such, several trials have been conducted looking at vitamin K2 supplementation in these

populations. In a recent study hemodialysis patients were supplemented with 2000µg Vitamin K2 MK-7 three times a week for 18 months without any adverse effects [50].

Given its potential role in reversing vascular calcification, many studies have investigated whether it can prevent cardiovascular disease. These trials have generally been conducted in small study groups and therefore occasionally report contradictory results. A currently ongoing trial 400 men between 65-74 years old are receiving either 720µg vitamin K2 MK-7 per day or placebo [51]. Though data from this trial is not yet available, there have been no major safety issues (Dr. Bergeland, personal communication).

To date there is no data on vitamin K supplementation during infectious diseases, with the exception of instances when it is given to correct bleeding disorders resulting from PT elongation, such as during Ebola virus disease [52].

6.4 Summary of known and potential risks and benefits

The European Food Safety Association reviewed guidelines for vitamin K intake in 2017. Given the paucity of information no tolerable upper intake level was set for vitamin K by the Scientific Committee on Food (SCF) [27]. Their review of the available literature did not find any adverse events thought to be associated with vitamin K toxicity at any studied dose in persons not using VKAs. Animal investigations have found no adverse effect after daily vitamin K administration of 2,000 mg/kg body weight for 30 days. Newborns are routinely administered supraphysiological doses of vitamin K parenterally (ranging from 0.2 mg/kg body weight to a 1 mg bolus dose), which have been demonstrated to induce mean/median serum vitamin K concentrations in the first week of life up to 1,000-fold higher than non-fasting adult 'normal' values. Furthermore, studies investigating different doses of parenteral vitamin K prophylaxis in infants looked the production of vitamin K metabolites, vitamin K recycling and vitamin K catabolic pathways and found that they were capable of metabolising large vitamin K doses [27].

Though these studies have generally been performed using vitamin K1, there is no evidence for toxicity of vitamin K2 MK-7 at dosages of 900µg/day or 2000µg 3 times per week, see section 6.3.

Given the poor appetite generally seen in COVID-19 patients and the fact that nutritional supplementation in Dutch hospitals does not contain significant amounts of vitamin K, there is little risk of patients inadvertently receiving higher dosages of vitamin K than intended.

6.5 Description and justification of route of administration and dosage

This is the first study to investigate the administration of vitamin K during COVID-19, and there is no dosage that has been previously shown to correct the deficiency. Available data indicates that the total vitamin K deficiency is quite severe, comparable to that seen in hemodialysis patients [26, 53].

Therefore, the current study will use the dosage 1000µg/day, which is comparable to the highest dosage previously studied in healthy persons and hemodialysis patients [46, 47, 50], see also section 6.4.

6.6 Dosages, dosage modifications and method of administration

Vitamin K2 MK-7 will be administered as tablets taken orally, either swallowed by the patient, or pulverized and administered as a suspension in water through a nasogastric tube in case a patient is unable to swallow the product.

The total daily dosage will be 1000µg/day, there will be no dosage modification during the trial.

6.7 Preparation and labelling of Investigational Product

In this study Vitamin K2 MK-7 will be administered orally according to the manufacturer's instructions. Boxes containing either 42 tablets of 333 µg vitamin K2 MK-7 or 42 tablets of placebo will be delivered by Kappa Biosciences AS to the pharmacy of the Canisius Wilhelmina Hospital. These will be labelled with numbers 1 to 40, see section 8.2 on randomization.

After inclusion into the study a patient will be issued a box of study product (either Vitamin K2 MK-7 or placebo). Upon issue the study nurse or investigator will fix a second label with the patient's name and hospital ID number to the box to avoid any chance of accidental study product switching if two patients enrolled in the study are staying in the same room.

If a patient is unable to swallow the product but has a nasogastric tube Vitamin K2 can be administered via this route. A nurse will pulverize the tablets and create a suspension in water that can be passed through the tube.

6.8 Drug accountability

The principal investigator must ensure that Vitamin K2 MK-7 and placebo are stored in a climate controlled environment according to the guidelines of the manufacturer. Accurate records must be maintained regarding the receipt of both Vitamin K2 MK-7 and placebo which include: date received, lot number, amount received.

Accurate records must also be maintained regarding issuing of Vitamin K2 MK-7 and placebo. This includes:

- Patient identification number
- Date and box code number
- Signature of the person issuing the trial medication

After the product has been issued treating nurse will be responsible for verifying the administration of the study product and noting this in the electronic patient system according to local hospital protocols.

7. NON-INVESTIGATIONAL PRODUCT

All subjects will be treated with at least a prophylactic dosage of heparin-based (heparin or any low-molecular weight heparin) anticoagulants according to local hospital protocols.

7.1 Name and description of non-investigational product(s)

Non-investigational medicinal product: heparin or low-molecular weight heparins (including enoxaparin, nadroparin and dalteparin)

7.2 Summary of findings from non-clinical studies

For more information on the structure and function of heparins and low-molecular weight heparins, see Farmacotherapeutisch Kompas.

In short, at therapeutic dosages heparin exerts its anticoagulant effect by inhibiting the function of activated coagulation factors X and II in an antithrombin (AT)-dependent manner [54]. In prophylactic dosages heparin only inhibits the function of activated factor X. Low-molecular weight heparins have a similar mechanism of action, but tend to have stronger inhibitory activity to factor X and weaker inhibitory activity towards factor II. Unlike nadroparin and dalteparin, enoxaparin also has weak activity against factor VII.

The therapeutic and prophylactic effects of all heparin-based anticoagulants are independent of vitamin K status.

7.3 Summary of findings from clinical studies

Low-molecular weight heparins are used extensively for thrombosis prophylaxis and treatment of thromboembolic diseases in patients admitted to the hospital and in out-patient settings. There is a broad body of data indicating their safety is comparable or superior to oral anticoagulant drugs [55, 56].

Recently a high number of thromboembolic complications were seen in COVID-19 patients, especially those admitted to the ICU [2]. Based on these findings national guidelines were revised to encourage use of thrombosis prophylaxis in all COVID-19 patients without significant contra-indications.

7.4 Summary of known and potential risks and benefits

The major risk of heparin or low-molecular weight heparins are bleeding. Current guidelines advise the use of prophylactic dosages of these anticoagulants in COVID-19 patients because the risk of major thromboembolic complications in these patients are thought to outweigh the risks of bleeding complications.

7.5 Description and justification of route of administration and dosage

All subjects will be treated with at least a prophylactic dosage of heparin-based (heparin or any low-molecular weight heparin) anticoagulants according to local hospital protocols.
Patients with an indication for therapeutic dosages of anticoagulants, including patients who upon admission use VKAs or DOACs, must be switched to a therapeutic dosage of heparin or any low-molecular weight heparin according to local hospital protocols and the discretion of the treating physician, prior to inclusion in the study.

7.6 Dosages, dosage modifications and method of administration

If patients develop thromboembolic complications during the study or are admitted to an ICU they can be treated with therapeutic dosages or higher prophylactic dosages of heparin-based anticoagulants, respectively, without being excluded from the study.
If during the course of this study local treatment guidelines on use of prophylactic or therapeutic anticoagulants in COVID-19 patients change, patients will be treated in accordance with those updated guidelines. If investigators believe the updated protocol increases the risks associated with participation in this clinical trial, this will be communicated to the reviewing Medical Ethical Committee as a protocol amendment.

7.7 Preparation and labelling of non-investigational product

This will be done according to local hospital protocols and specific manufacturer's instructions.

7.8 Drug accountability

Drug administration will be recorded by the treating nurse in the electronic patient system according to local hospital protocols.

8. METHODS

8.1 Study parameters/endpoints

8.1.1 Main study parameters/endpoints

- Plasma desmosine levels before and during vitamin K supplementation in intervention versus control patients
- Plasma dp-uc MGP levels before and during vitamin K supplementation within the intervention group and in intervention versus control patients

8.1.2 Secondary study parameters/endpoints

- Difference between the number of grade 3 and grade 4 adverse events between the intervention and control group, with special attention for: progression of respiratory insufficiency, thrombotic events, pulmonary embolism or deep venous thrombosis, bleeding, renal insufficiency, cardiac decompensation, liver enzyme abdnormalities and/or liver failure.
- Changes in serum PIVKA-II levels during COVID-19 with or without vitamin K supplementation

8.1.3 Exploratory study parameters

- Serum (undercarboxylated) protein S levels before and after vitamin K supplementation
- Incidence of respiratory failure defined as either intubation and mechanical ventilation or death (with respiratory failure as a likely direct or indirect cause of death) in the intervention versus control group
- Change in disease severity as measured by low-dose chest CT at baseline and on day 5 in intervention versus control groups
- Plasma levels of active MGP before and after vitamin K supplementation
- Normative APC sensitivity ratio and prothrombin (Echis Carinato test) and TFPI concentration before and after vitamin K supplementation
- Inflammatory parameters including CRP, d-dimer, ferritine, IL-6, TNF-α, IFN-γ and s-IL-2r as measured by ELISA or Multiplex in both intervention and control groups
- Other markers related to the pathogenesis, susceptibility and severity of COVID-19 in both intervention and control groups
- Vitamin K epoxide reductase complex subunit 1 (VKORC-1) gene polymorphisms and vitamin D receptor gene polymorphisms

8.2 Randomisation, blinding and treatment allocation

Forty patients will be allocated receive either vitamin K2 MK-7 supplementation or placebo in a double-blind manner.

20 boxes containing tablets of Vitamin K2 MK-7 and 20 boxes containing placebo tablets will be manufactured by Kappa Biosciences AS and randomly assigned numbers from 1 to 40 as follows.

Prior to the study start a treatment randomization list will be generated using the Microsoft Excel command ASELECTUSSEN (0,1000). Someone from the Canisius Wilhemina Hospital pharmacy will be responsible for performing the randomization and for assigning treatment numbers to either the intervention or placebo group. A second pharmacy employee, not involved in the assignment, will check to see if randomization is done correctly. Using Excel, the treatment numbers 1 to 40 will be linked to a randomization number between 0 and 1000. The treatment numbers with the twenty lowest randomization numbers will be assigned the vitamin K2 MK-7 boxes and treatment numbers with the twenty highest randomization numbers will be assigned to the placebo boxes. If two identical numbers are produced, the whole procedure is repeated. The master randomization list is kept at the Canisius Wilhelmina Hospital pharmacy.

Using this procedure the CWZ pharmacy will generate a list of treatment numbers 1 to 40 followed by either ‘Vitamin K2 MK-7’ or ‘placebo’ and send this list via a secure e-mail to the representative of Kappa Biosciences AS delegated to perform the assignment of treatment numbers to the product boxes. Kappa Biosciences AS will label the intervention and placebo boxes according to the list generated by the pharmacy.

Once a patient is included into the study they will be assigned to the lowest a treatment number still available at the trial site. Treatment number will be registered on the drug accountability log (section 6.8) and in the eCRF.

This is a double blind study: patients, treating physicians, investigators and those performing laboratory analysis and other outcome assessments will be blinded to the identity of study groups until all patients complete primary endpoint assessments (occurring on the last day of treatment of the last patient).

The randomization list is kept by the CWZ pharmacy. A patient’s treatment allocation can be deblinded by the CWZ pharmacy at the request of the treating physician by one of the investigators only after discussion with the principle investigator. The allocation will only be deblinded if it is absolutely necessary for the safety of the patient. If the code is broken it will be documented.

8.3 Study procedures

8.3.1 Baseline screening

Subjects who sign informed consent will be eligible for participation. The treating physician, if need be in collaboration with study staff, will confirm the patient meets the in- and exclusion criteria. Namely, screening will include evaluation of SARS-CoV-2 PCR results, vital signs, concomitant medication and known allergies.

8.3.2 Study medication

Trial medication will be delivered by the local pharmacy coded only with the trial identifier and the patient study code (see also section 6.8). Directly after inclusion (defined as study day 1) in the study patients will take either 3 tablets containing 333mcg K2 each (for a total dose of 1000mcg) or 3 matched placebo tablets. Patients will continue taking either 1000mcg Vitamin K2 MK-7 once a day or matched placebo for up to 14 days. Treatment will be discontinued at discharge.

8.3.3 Follow-up of adverse events

Adverse events will be monitored until discontinuation of treatment, at 28 days post inclusion mortality will be assessed.

When recording adverse events special attention will be paid to progression of respiratory insufficiency, thrombotic events, pulmonary embolism or deep venous thrombosis and bleeding. Toxicities will be scored according to the most recent version of the NCI Common Terminology Criteria for Adverse Events (CTCAE) version 5 (see Appendix).

The treating physician will determine whether adverse events require additional testing, such as, for example, pulmonary CT angiography in the case of suspected pulmonary embolism or coagulation parameters in the case of bleeding.

8.3.4 Vital signs

Vital signs including body temperature, respiration rate, oxygen saturation measured by pulse oximetry, blood pressure and pulse measurements will be determined and recorded daily as part of routine clinical practice at least once a day.

Systolic and diastolic blood pressure will be measured while the subject is lying down or sitting with back supported, using an automated validated device, with an appropriately sized cuff. In case the cuff sizes available are not large enough for the subject's arm circumference, a sphygmomanometer with an appropriately sized cuff may be used.

Temperature will be measured according to local practice, consistently throughout the study. The thermometer used should have a precision of 0.1ºC. The same route should be used throughout the study.

If vital signs are out-of-range, the treating nurse of physician may obtain additional readings at their discretion.

8.3.5 Clinical laboratory measurements

At baseline (within 24 hours of admission) and subsequently three times per week, the following laboratory tests are performed: full blood count, electrolytes (sodium, potassium, calcium), creatinine, liver enzymes (bilirubin, ALAT, ASAT and LDH), c-reactive protein (CRP) and albumin. D-dimer will be performed at all follow-up time points (though not at baseline).
Arterial blood gas can be performed at the discretion of the treating physician but will not be part of routine measurements.
Performing additional laboratory analyses such as urinalysis, d-dimer, or cardiac enzymes in the context of standard patient care is at the discretion of the treating physician.

8.3.6 Dp-ucMGP

At baseline (within 24 hours of inclusion) and subsequently three times per week, EDTA plasma will be frozen at -80 degrees Celsius for retrospective determination of dp-ucMGP. Circulating dp-ucMGP levels will be determined using the commercially available IVD CE marked chemiluminescent InaKif MGP assay on the IDS-iSYS system (IDS, Boldon, UK). 50 µL of patient sample or calibrators are incubated with magnetic particles coated with murine monoclonal dpMGP antibody, an acridinium labelled murine monoclonal ucMGP antibody and assay buffer. The magnetic particles are captured using a magnet and a wash step performed to remove any unbound analyte. Trigger reagents are added, the resulting light emitted by the acridinium label is directly proportional to the concentration of dp-ucMGP in the sample. The within-run and total precision of this assay is 0.8 – 6.2% and 3.0 – 8.2%, respectively. The assay measuring range is between 300 – 12,000 pmol/L and was previously found to be linear up to 11,651 pmol/L. Dp-ucMGP values below 500 pmol/L are considered to be in the normal healthy range.

8.3.7 PIVKA-II

At baseline (within 24 hours of inclusion) and subsequently three times per week, EDTA plasma will be frozen at -80 degrees Celsius for retrospective determination of dp-ucMGP. Plasma PIVKA-II concentrations will be measured with ELISA using a commercially available kit from Roche Diagnostics (Elecsys® PIVKA-II).

8.3.8 Desmosine

The rate of elastin degradation will be quantified by measuring plasma (p)DES levels. At baseline (within 24 hours of inclusion) and subsequently three times per week, EDTA plasma

will be frozen at -80 degrees Celsius for retrospective determination of pDES. Subjects with the highest pDES are assumed to have the highest rates of elastin degradation. Isodesmosine and desmosine fractions will be measured separately by liquid chromatography-tandem mass spectrometry as previously described using deuterium-labelled desmosine as internal standard [57, 58].

8.3.9 Exploratory laboratory measurements

At every time point extra EDTA plasma, citrate plasma an serum will be aliquotted and frozen at -80 degrees Celsius for retrospective analysis of exploratory outcomes. These can include measurements related to protein S and coagulation activity, including levels of (undercarboxylated) protein S, normative APC sensitivity ratio and prothrombin (Echis Carinato test), TFPI concentration and vitamin D levels.

Levels of circulating active MGP can be measured by ELISA. Furthermore, levels of inflammatory markers parameters including CRP, d-dimer, ferritine, IL-6, TNF-α, IFN-γ and s-IL-2r can measured by ELISA or Multiplex assay.

VKORC1 polymorphism can be determined as follows. High Pure PCR Template Preparation Kit (Roche Diagnostics, Mannheim, Germany) will be used for isolation of DNA, according to the manufacturer's instructions. For genotyping the C1173T (rs 9934438) and G-1639A (rs 9923231) single nucleotide polymorphisms of the VKORC1 gene, real-time PCR Fluorescence Resonance Energy Transfer (FRET) analyses will be performed. FRET LightMix® assays (cat.-no 40-0302-16, TIB MOLBIOL, Berlin, Germany) on the LightCycler® (Roche Diagnostics) will be used, according to the manufacturer's protocols. The assay consists of a duplex reaction measuring the melting curves of the used specific fluorescent probes in two different channels, each with a distinct wavelength. With each run positive (heterozygote, provided with the kit) and negative controls will be determined.

8.3.10 Chest Computer Tomography (CT) scans

Computed tomography (CT) is performed at baseline and on day 5 after study start for assessing pulmonary involvement score (Dofferhoff *et al.* CID 2020)). If patients are discharged before day 5 the second CT scan is made prior to discharge. Severity of COVID-19 pneumonia will be quantified and expressed as volume percentage of lung involvement on low dose chest CT by a specialized chest radiologist at University Medical Center Utrecht (UMCU) as described below.

Thin slice CT scans are acquired by using a Philips Ingenuity multi-detector row scanner (Philips Healthcare). CT images of 1-mm thickness are reconstructed by using iterative model-based reconstruction in the axial plane.

Quantitative measurements of the volume of ground glass and consolidation will be undertaken using the Intellispace Portal (COPD package, Intellispace version 10, Philips Healthcare). In the software, first the lungs are segmented from the chest wall and major vessels and main bronchi. Manual adjustments are implemented where required by a board-certified chest radiologist. Subsequently, the lung voxels are counted to derive a total lung volume in milliliters. Diseased lung tissue is defined as those voxels with an attenuation of Hounsfield Units (HU) > -700. The abnormal voxels are expressed as a percentage diseased lung of the total volume. HU value at the 85th percentile will be used.

8.3.11 Vitamin K epoxide reductase complex subunit 1 (VKORC-1) gene polymorphisms

At one time point during the trial we will take a DNA mouth swab from each patient for determination of the single-nucleotide polymorphism (SNP) C1173T on the VKORC1 gene, using the Roche Diagnostics LightCycler® (Roche Applied Science, Indianapolis, IN) diagnostic test.

8.3.12 Study flow chart

	Inclusion	Hospital admission		Follow-up
Study day	1	2-14[6]	15-27[6]	28
Eligibility criteria + informed consent	X			
Collection of demographic data and medical history	X			
Physical examination and vital signs[1]	X	X	X	
Vitamin K2 MK-7 or placebo treatment	X	X		
Collecting (serious) adverse events		as necessary		
Collecting adverse events		X		X
Laboratory testing[2,3]	X[7]	X	X	
dp-ucMGP, PIVKA-II, desmosine[2,4]	X[7]	X	X	
Plasma and serum collection for exploratory endpoints[2,4]	X[7]	X	X	
CT scan	X[8]	X[5]		

[1] Vital signs are collected at least 3 times per day until discharge, physical examination is performed as necessary

[2] Blood is collected three times per week while the patient is admitted to the hospital

[3] Full blood count, electrolytes (sodium, potassium, calcium), creatinine, liver enzymes (bilirubin, ALAT, ASAT and LDH), c-reactive protein (CRP) and albumin; d-dimer will be determined at all follow-up time points (though not at baseline)

[4] Analysis performed retrospectively

[5] Performed at baseline and on day 5, if patient is discharged before day 5 a CT scan is performed prior to discharge, if the CT scan cannot be made on day 5 it is made up to a maximum of 24 hours after day 5

[6] Number of study days dependent on discharge

[7] Or in the previous 24 hours

[8] Or in the previous 52 hours

8.4 Withdrawal of individual subjects

Subjects can leave the study at any time for any reason if they wish to do so without any consequences. The investigator can decide to withdraw a subject from the study for urgent medical reasons or the following reasons:

- Refusal of patient to continue protocol treatment
- No compliance of the patient: patient is unable or unwilling to adhere to the treatment schedule and/or procedures required by the protocol

Patients who are withdrawn from protocol treatment will receive medical care according to local hospital practice.

If a patient states that he or she withdraws their consent to participate in the trial, the investigator should attempt to verify the patient’s intent and their reason for withdrawal, and record this in the patient’s medical file.

The patient can refuse further treatment and/or procedures according to protocol, while still consenting with further follow up data collection. The patient can refuse further treatment and/or procedures according to protocol and withdraw consent for further follow up data collection.

For patients who are lost to follow-up (i.e. those subjects whose status at 28 days post treatment is unclear because they cannot be contacted without having stated an intention to withdraw), extensive effort (i.e. documented phone calls and e-mails) will be undertaken to determine his or her health status. The investigator should show "due diligence" by documenting in the source documents steps taken to contact the patient.

8.4.1 Specific criteria for withdrawal

Patients should be withdrawn from protocol treatment if any of the following criteria for withdrawal are met:

- Patient is not eligible to participate in the study in hindsight
- - The occurrence of an adverse event preventing further treatment Patient becomes dialysis-dependent

.

8.5 Replacement of individual subjects after withdrawal

If a patient withdraws or is withdrawn before he or she has received the first treatment (vitamin K2 or placebo), he/she will be replaced with another suitable patient.

8.6 Follow-up of subjects withdrawn from treatment

After discharge the investigator will follow-up the patients status as alive or deceased after 28 days after study intervention. If the patient is seen in outpatient clinic by a treating physician this information may be gathered from the patient's medical file. Alternatively, an investigator can call the patient's primary care physician or the patient at home to ascertain this information.

8.7 Premature termination of the study

The sponsor may decide to terminate the study prematurely based on the following criteria:

- There is evidence of an unacceptable risk for study patients (i.e. safety issue);
- There is reason to conclude that continuation of the study cannot serve a scientific purpose following confirmation of the SMC; or
- The SMC recommends to end the trial based on viable arguments other than described above.

The sponsor will promptly notify all concerned investigators, the Ethics Committee(s) and the regulatory authorities of the decision to terminate the study. The sponsor will provide information regarding the time lines of study termination and instructions regarding treatment and data collection of enrolled patients.

9. SAFETY REPORTING

9.1 Temporary halt for reasons of subject safety

In accordance with section 10, subsection 4, of the WMO, the sponsor will suspend the study if there is sufficient ground that continuation of the study will jeopardise subject health or safety. The sponsor will notify the accredited METC without undue delay of a temporary halt including the reason for such an action. The study will be suspended pending a further positive decision by the accredited METC. The investigator will take care that all subjects are kept informed.

9.2 AEs and SAEs

9.2.1 Adverse events (AEs)

Adverse events are defined as any undesirable experience occurring to a subject during the study, whether or not considered related to the experimental intervention. All adverse events reported spontaneously by the subject or observed by the treating physician, the investigator or the research nurses are recorded into the patient's medical files.
Given the expected large number of adverse events occurring in patients admitted with COVID-19, only grade 3 and 4 adverse events will be reported in the Electronic Case Report Forms (eCRFs). Grade 3 and 4 adverse events will be recorded if they begin or become more severe between the time of the first treatment and discontinuation of treatment.
The severity of any adverse events, including any lab abnormality, will be determined by using the Common Terminology Criteria for Adverse Events, version 5 (NCI CTAE, appendix 1). The criteria are also available online at http://ctep.cancer.gov.
In any case where the NCI CTCAE criteria do not apply, severity should be defined according to the following criteria:

Mild (grade 1): awareness of symptoms that are easily tolerated and do not interfere with usual daily activity
Moderate (grade 2): discomfort that interferes with or limits usual daily activity
Severe (grade 3): disabling, with subsequent inability to perform usual daily activity, resulting in absence or required bed rest
Life threatening (grade 4): immediate risk of death from the reaction as it occurred

9.2.2 Serious adverse events (SAEs)

A serious adverse event is any untoward medical occurrence or effect that:

- results in death;
- is life threatening (at the time of the event);
- requires hospitalisation or prolongation of existing inpatients' hospitalisation;
- results in persistent or significant disability or incapacity;
- is a congenital anomaly or birth defect; or
- any other important medical event that did not result in any of the outcomes listed above due to medical or surgical intervention but could have been based upon appropriate judgement by the investigator.

An elective hospital admission or outpatient visit will not be considered as a serious adverse event.

The investigator will report all SAEs to the sponsor without undue delay after obtaining knowledge of the events.
As symptoms in COVID-19 patients are often progressive after admission, an adverse event that prolongs hospitalization will only be considered a serious adverse events if, according to the investigator, it falls outside the natural progression of COVID-19.
Serious Adverse Events (SAEs) will be reported from the first study-related procedure until discharge from the hospital (at which time treatment will be discontinued) or until study day 28 if the patient is still admitted at that time. SAEs will be followed up for every patient on study day 28.

Toetsing Online

The investigator will report all SAEs to the sponsor without undue delay after obtaining knowledge of the events. The sponsor will report the SAEs through the web portal *ToetsingOnline* to the accredited METC that approved the protocol, within 7 days of first knowledge for SAEs that result in death or are life threatening followed by a period of maximum of 8 days to complete the initial preliminary report. All other SAEs will be reported within a period of maximum 15 days after the sponsor has first knowledge of the serious adverse events.

9.3 Annual safety report

In addition to the expedited reporting of SAEs, the sponsor will submit, once a year throughout the clinical trial, a safety report to the accredited METC, competent authority, and competent authorities of the concerned Member States.
This safety report consists of:

- a list of all serious adverse events;
- a report concerning the safety of the subjects, consisting of a complete safety analysis and an evaluation of the balance between the efficacy and the harmfulness of the medicine under investigation.

9.4 Follow-up of (serious) adverse events

9.4.1 (Serious) adverse event data collection

Safety assessments will be performed and recorded by the investigators. All adverse events/reactions (solicited and unsolicited), noted by the investigators will be accurately documented in the case report form by the investigators. For each event/reaction the following details will be recorded:

1. Description of the event(s)/reaction(s)
2. Date and time of occurrence
3. Duration
4. Intensity
5. Relationship with the intervention
6. Action taken, including treatment
7. Outcome

9.4.2 Assessment of causality

The investigators are obligated to assess the relationship between study procedures and the occurrence of each AE/SAE. The investigators will use clinical judgment to determine the relationship. Alternative causes, such as natural history of COVID-19 or any underlying diseases, concomitant therapy, other risk factors and the temporal relationship of the event to the challenge will be considered and investigated. The relationship of the adverse event with the study procedures will be categorized as:

Probable	An adverse event that follows a reasonable temporal sequence from the challenge procedure and cannot be reasonably explained by the known characteristics of the subject's clinical state.
Possible	An adverse event for which insufficient information exists to exclude that the event is related to the study procedure.
Not related	An event for which sufficient information exists to indicate that the aetiology is unrelated either because of the temporal sequence of events or because of the subject's clinical state or other therapies.

9.4.3 Follow-up of (serious) adverse events

All adverse events will be followed clinically until they have been resolved, or until a stable situation has been reached. Depending on the event, follow up may require additional tests or medical procedures as indicated, and/or referral to the general physician or a medical specialist.

SAEs need to be reported till end of study within the Netherlands, as defined in the protocol as treatment discontinuation for the last patient.

9.5 Safety Monitoring Committee

An independent Safety Monitoring Committee (SMC) will be appointed, including 3 individuals. Their main responsibility will be an interim analysis of all serious adverse events and occurred thromboembolisms, making the decision to halting further study procedures. A safety report including a list of all reported serious adverse events and thromboembolisms will be prepared for review by the SMC after the first 20 patients have completed follow-up and at the end of the study.

The advice(s) of the SMC will be sent to the sponsor of the study. Should the sponsor decide not to fully implement the advice of the SMC, the sponsor will send the advice to the METC that assessed the study, including a note to substantiate why (part of) the advice of the SMC will not be followed.

9.5.1 Review of safety data by the safety monitor and SMC

A safety report including information on all reported serious adverse events and thromboembolisms will be compiled after the twentieth patient has reached the fourteenth day of the study (discontinuation of treatment) and at the end of the study. These reports will be prepared in a blinded manner by a clinical investigator and sent to the SMC and all principle investigators. During a closed session, the SMC will be able to de-blind the information prior to their review.

The responsibilities of the SMC are described in the SMC Charter. In short, the SMC will evaluate the number and nature of any SAEs and thromboembolisms that occurred during the study. In the case of statistically significant increase in the number of SAEs and thromboembolisms in the intervention group, compared to the control group, the SMC will advise the sponsor to halt the study pending further review.

The advice(s) of the SMC will only be communicated to the METC when the sponsor does not follow this. With this notification a statement will be included indicating whether the advice will be followed.

9.5.2 Safety stopping rules

The study may be placed on safety hold for the following reasons:

- On advice of one of the principle investigators
- On advice of the SMC
- On advice of the METC that assessed the study

The safety monitor, METC, or investigators may decide to put the study on hold based on adverse events and amount of thromboembolisms, pending discussion with the safety monitor, SMC, METC and investigators. In addition, a principle investigator can always decide based on characteristics, duration and severity of signs/symptoms to treat and stop the trial for individual cases. In such cases, the principle investigator will alert the representative of the sponsor. If the METC has recommended safety hold, re-initiation of the study will require METC concurrence. The METC will be informed of a safety hold by the sponsor. Following discussion, it may be decided to terminate the study.

10. STATISTICAL ANALYSIS

All patients who receive at least one study treatment (the initial dose vitamin K2 or placebo) will be included in the intention-to-treat analysis.

10.1 Primary study parameters

The primary outcome of difference in dp-ucMGP (log-transformed values) will be assessed by comparing samples pre- and post-treatment using a matched-samples t-test and between groups using an independent samples t-test. Desmosine (log-transformed values) will be compared between the intervention and control groups using an independent samples t-test. Additionally, ANCOVA testing can be performed for both outcome measurements.

10.2 Secondary and exploratory study parameters

Adverse events will be compared by tabulating all grade 3 and 4 adverse events for each patient and calculating the proportion of in each group. The incidence of complications will be compared between groups with the chi-square test or Fishers exact test.

In the exploratory analyses, we will assess differences by comparing mean values between the groups using either a two-tailed student’s t-test or non-parametric equivalents, paired if pre-treatment values are compared with post-treatment values, unpaired if comparisons are made between groups. For discrete variables (e.g. the number of positive assays), the chi-squared test or Fisher’s exact test will be used (two-tailed).

As an exploratory analysis data will also be visualized on an individual patient level to evaluate the progression or correction of vitamin K deficiency in relationship to other parameters associated with COVID-19 disease severity.

10.3 Interim analysis

There will be no interim data analysis. Fifty percent of the study patients, 10 intervention and 10 controls, has insufficient power to conduct a classical interim analysis to evaluate either the primary or secondary endpoints. Instead, the SMC will conduct a safety analysis that will focus on identifying any serious negative effect of the study intervention on patient safety.

The SMC will have access to the unblinded patient data for all serious adverse events and thromboembolisms that occurred during the trial. If a significantly higher number of SAEs or thromboembolisms occurred in the intervention group compared to the control group, the SMC will advise the sponsor to place the study on hold pending a more extensive review. In the case of such a safety hold, the trial will only be restarted after discussion with the METC that assessed the study.

11. ETHICAL CONSIDERATIONS

11.1 Regulation statement

This study will be conducted according to the principles of the Declaration of Helsinki (Brazil 2013, www.wma.net) and in accordance with the Medical Research Involving Human Subjects Act (WMO), GCP and the GDPR.
The investigator will be thoroughly familiar with the appropriate use of the study drug as described in the protocol and Investigator's Brochure. Essential clinical documents will be maintained to demonstrate the validity of the study and the integrity of the data collected. The Sponsor's Trial Master File (TMF) will be maintained at the sponsor site (CWZ). Each participating site will maintain an investigator site file (ISF). This study file will be established at the beginning of the study.

The ethics committee(s) will review all appropriate study documentation in order to safeguard the rights, safety and well-being of the patients. The study will only be conducted at sites where ethics approval has been obtained. The investigator will provide the relevant ethics committee(s) with the final version of the protocol, patient information and consent forms, any other written information given to patients, safety updates, annual progress reports, and any revisions to the study protocol or any other trial documentation

Protocol compliance

The investigator will conduct the study in compliance with the protocol given approval/favorable opinion by the ethics committee(s), the appropriate Regulatory Authority(ies) and, if required, institutional department(s) such as Research & Development Department. Changes to the protocol will require approval from the sponsor and written ethics committee approval/favorable opinion prior to implementation, except when the modification is needed to eliminate an immediate hazard(s) to patients. The ethics committee(s) may provide, if applicable Regulatory Authority(ies) permit, expedited review and approval/favorable opinion for minor change(s) in ongoing studies that have the approval /favorable opinion of the ethics committee(s). The investigator will submit all protocol modifications (non- compliance) to the regulatory authority(ies) in accordance with the governing regulations. Any deviations from the protocol must be fully documented in the source documents.

11.2 Recruitment and consent

The method of obtaining and documenting the informed consent and the contents of the consent will comply with ICH-GCP and all applicable regulatory requirement(s):

Patients will be informed about the study by their treating physician. After initial information, patients will be given the informed consent form and additional information about the study can be provided by the study nurse or researcher.
A properly signed and personally dated informed consent form is required for each patient before any trial specific procedure. After the study has been fully explained the patient should be given ample time to read the consent forms and ask questions.
Written informed consent will always be obtained from the patient prior to study participation. The informed consent process should be recorded in source documents (date of information and consent, parties present).
The investigator is responsible for checking entries made by the patient on the consent form, and to request correction immediately in case of missing, illegible or incorrect dates. The person taking the patient consent should sign and date both consent forms to confirm he/she provided information to the subject.

The Informed Consent form will be updated by the investigator whenever important new information becomes available that may be relevant to subject’s consent. This may be a result of amendments to the protocol, new information regarding the trial medication alternative treatments. Revised versions must be approved by the relevant ethics committee(s).
The revised consent form must be signed by subjects who are entered in the trial and not yet completed, if these changes are relevant to the subject’s willingness to continue participation. In particular, if the consent form is updated with new safety information, a new version of the informed consent form must be provided to all subjects still participating in the trial in a timely manner as soon as written ethics approval is obtained. Patient withdrawal of consent from the study should be explicitly documented in the source documents.

After the patient signs informed consent the study physician will also inform their primary contact person designated in their patient file.

11.3 Benefits and risks assessment, group relatedness

This is a double blind therapeutic randomized safety clinical trial. Half of the patients will receive the intervention which may be beneficial for the trial subject. There is a potential benefit for participants in this study if vitamin K2 is shown to help prevent complications from COVID-19. However, there is no preliminary causal evidence for this relationship.

Risks for participants are related to three interventions: 1) taking vitamin K2 MK-7, 2) repeated blood draws, 3) extra low-densitiy CT scan(s). To date no adverse side-effects from

vitamin K2 MK-7 supplementation have been described in persons who do not use vitamin K antagonists (VKAs). Blood will be drawn by venapuncture or through an intravenous cannula at regular time points as part of routine patient care. In study participants 25ml extra blood will be three times a week during admission. This volume is not expected to have consequences for the health of the patients.
Finally, in participants one or two extra low dose CTs will be made outside routine care for COVID-19. The risks of this are expected to be minimal as the radiation exposure of a single low-dose CT is low.

11.4 Compensation for injury

The sponsor/investigator has a liability insurance which is in accordance with article 7 of the WMO.
The sponsor (also) has an insurance which is in accordance with the legal requirements in the Netherlands (Article 7 WMO). This insurance provides cover for damage to research subjects through injury or death caused by the study.
The insurance applies to the unexpected damage that becomes apparent during the study or within 4 years after the end of the study.

12. ADMINISTRATIVE ASPECTS, MONITORING AND PUBLICATION

12.1 Handling and storage of data and documents

Data and documents will be controlled and processed conform the EU General Data Protection Regulation (GDPR) and the Dutch Act on Implementation of the General Data Protection Regulatio. (in Dutch: Uitvoeringswet AVG, UAVG).

In order to maintain patient privacy, all data capture records, study drug accountability records, study reports and communications will identify the patient by the assigned patient number. The full patient name should never be used in any correspondence the case report forms.

12.2 Case Report Forms

Data will be collected on electronic Case Report Forms (CRF) to document eligibility, safety and efficacy parameters, compliance to treatment schedules and parameters necessary to evaluate the study endpoints. Data collected on the CRF are derived from the protocol and will include at least:

- Inclusion and exclusion criteria
- Baseline status of patient including medical history and stage of disease;
- Timing and dosage of protocol treatment;
- Baseline concomitant diseases and adverse events;
- Parameters for response evaluation;
- Any other parameters necessary to evaluate the study endpoints;
- Survival status of patient;

The e-CRF will be completed on site by the local investigator or sub-investigator or an authorized staff member. The CRF must be signed by the local investigator or sub-investigator upon completion by means of an electronic signature. All CRF entries must be based on source documents.

12.3 Subject confidentiality

Each patient is assigned a unique patient study number at enrolment. In trial documents the patient's identity is coded by patient study number as assigned at enrolment. The local investigator will keep a subject enrolment and identification log that contains the key to the code, i.e. a record of the personal identification data linked to each patient study number. This record is filed at the investigational site and should only be accessed by the investigator and the supporting hospital staff, and by representatives of the sponsor or a regulatory agency for the purpose of monitoring visits or audits and inspections.

Only encoded data may be shared with other study groups for research purposes.

12.4 Filing of essential documents

It is the responsibility of the principal investigator at the study center to keep all essential documents relating to the trial for at least 15 years after the completion or premature termination of the clinical trial. Essential Documents are those documents that permit evaluation of the conduct of a trial and the quality of the data produced and show whether the institution complied with the principles and guidelines of good clinical practice. The essential documents may be subject to, and should be available for, audit by the sponsor's auditor and inspection by the regulatory authority(ies).

The investigator should file all essential documents relevant to the conduct of the trial on site. The sponsor will file all essential documents relevant to the overall conduct of the trial. Essential documents should be filed in such a manner that they are protected from accidental loss and can be easily retrieved for review. The archived data can be kept in electronic form, provided the protocol, ethical and government approvals, together with all other documents concerning the study, including any audit and inspection certificates are all to be kept as part of the trial master reference file. All data about serious adverse events and occurred thromboembolisms also need to be kept in this trial master file.

The medical files of patients enrolled into the trial must be kept in accordance with national legislation and for the maximum period of time permitted by the institution.

12.5 Storage of samples

Storage of biological samples on site is subject to the site's guidelines; samples may be labeled with the patients identifying information (e.g. name, hospital record number). Samples that are shipped to another facility for a purpose as described in this protocol or for additional scientific research, should be stripped from any identifying information and labeled with a the trial name and individual patient's study code.

12.6 Monitoring and Quality Assurance

Before study initiation, the protocol and eCRFs together with relevant SOPs will be reviewed by the sponsor, the investigators and their staff. During and after completion of the study, the data monitor will visit each site to check the completeness of records, the accuracy of entries on the eCRFs, the adherence to the protocol and to Good Clinical Practice, the progress of enrolment, and to ensure that Vitamin K2 and placebo are being dispensed and accounted for according to protocol.

The site will maintain source documents for each subject in the study consistent with hospital guidelines, including admission and visit notes containing demographic and medical

information, laboratory data, and the results of any other tests or assessments. Data concerning the primary outcome is drawn from these source documents and recorded in the eCRFs. The investigator will also keep the original informed consent form signed by the subject (a signed copy is given to the subject).
The investigator will give the data monitor access to all relevant source documents to confirm their consistency with the eCRF entries. Though there is are no indications of possible adverse effects from Vitamin K2 MK-7 supplementation, this study has been classified as 'middle risk' according to the NFU risk classification system based on the vulnerability of the study population. The monitor will perform full verification for the presence of informed consent, adherence to the inclusion/exclusion criteria and documentation of SAEs. The recording of data that will be used for all primary and safety variables will be assessed for 10% of included subject (i.e. 4 subjects).

12.7 Amendments

Amendments are changes made to the research after a favourable opinion by the accredited METC has been given. All amendments will be notified to the METC that gave a favourable opinion. A 'substantial amendment' is defined as an amendment to the terms of the METC application, or to the protocol or any other supporting documentation, that is likely to affect to a significant degree:

- the safety or physical or mental integrity of the subjects of the trial;
- the scientific value of the trial;
- the conduct or management of the trial; or
- the quality or safety of any intervention used in the trial.

All substantial amendments will be notified to the METC and to the competent authority. Non-substantial amendments will not be notified to the accredited METC and the competent authority, but will be recorded and filed by the sponsor.

12.8 Annual progress report

The sponsor will submit a summary of the progress of the trial to the accredited METC once a year. Information will be provided on the date of inclusion of the first subject, numbers of subjects included and numbers of subjects that have completed the trial, serious adverse events/ serious adverse reactions, other problems, and amendments.

12.9 Temporary halt and (prematurely) end of study report

The sponsor will notify the accredited METC and the competent authority of the end of the study within a period of 90 days. The end of the study is defined as the last day of follow-up for the last patient (defined as discontinuation of treatment).
In case the study is ended prematurely, the sponsor will notify the accredited METC within 15 days, including the reasons for the premature termination.
Within one year after the end of the study, the investigator/sponsor will submit a final study report with the results of the study, including any publications/abstracts of the study, to the accredited METC.

12.10 Public disclosure and publication policy

This trial will be registered in clinicaltrials.gov prior to study start. The final report will be prepared by the investigators at the Canisius Wilhelmina Hospital. It will be signed by the project leader and on behalf of the sponsor. The investigators will make every effort to publish the results in a peer-reviewed journal.

13. STRUCTURED RISK ANALYSIS

13.1 Potential issues of concern

a. Level of knowledge about mechanism of action

Vitamin K2 in the form of menaquinone-7 is approved as a nutritional supplement in the European Union and is readily available in stores in The Netherlands.

The activity of vitamin K in general, including Vitamin K2 MK-7 specifically, has been studied both *in vitro* and *in vivo*. Its role as a cofactor for the enzyme γ-glutamate carboxylase (GGCX) during the reaction in which it carboxylates glutamate residues into γ-carboxyglutamate (Gla) has been described extensively [28]. Vitamin K has been extensively studied for its critical role in activating hepatic coagulation factors II, VII, IX and X, protecting against bleeding disorders that are seen in patients with a severe deficiency [59]. The mechanisms by which vitamin K contributes to bone health by acting as a cofactor for the carboxylation of osteocalcin has also been explored in many human studies [49].

Vitamin K is crucial for the activation of MGP via the same mechanism of vitamin-K-dependent carboxylation by GGCX. The importance of MGP to preventing vascular calcifications has been highlighted by many cohort studies associating increased levels of dp-ucMGP (uncarboxylated MGP) with increased vascular calcifications. There are also multiple studies showing that vitamin K supplementation has a favorable effect on vascular calcification, though large scale studies are still ongoing [25, 51]. It has been hypothesized that vitamin K protects pulmonary elastic fibers through a similar mechanism, but this remains to be definitively established [23].

Less is known about the role of vitamin K in anticoagulation pathways. Through the same mechanism as procoagulation factors, vitamin K is required for the carboxylation of anticoagulant protein C and protein S [60, 61]. It has been theorized that an initial vitamin K insufficiency would result in deficient activation of endothelial protein S before causing a decrease in carboxylated prothrombin. This would explain the seemingly paradoxical increase of thrombosis risk in the first week of treatment with VKAs [18, 62].

b. Previous exposure of human beings with the test product(s) and/or products with a similar biological mechanism

Vitamin K2 in the form of menaquinone-7 is approved as a nutritional supplement in the European Union and is readily available in stores in The Netherlands.

Pharmacokinetics of vitamin K2 MK-7 have been previously examined in humans [25, 46, 47]. Studies both in healthy volunteers and hemodialysis patients have used dosages of vitamin K2 MK-7 similar to the dosage in this trial without any safety concerns, including thrombotic complications [46, 47, 49-51].

c. Can the primary or secondary mechanism be induced in animals and/or in *ex-vivo* human cell material?

In a previous study we have used available biobanks of plasma from COVID-19 patients to establish much of the correlations between vitamin K status, MGP, elastic fiber degradation and clinical outcome.

Though SARS-CoV-2 can infect other species, robust animal disease models of COVID-19 are lacking. *Ex-vivo* human cell cultures can and have been used to study the activity of vitamin K on a molecular basis, but these cannot capture the complexities of pharmacokinetics and tissue distribution that will be studied in this clinical trial.

d. Selectivity of the mechanism to target tissue in animals and/or human beings

The activity of vitamin K is not tissue specific. The triage theory posits that in times of vitamin K insufficiency it is first used to activate intrahepatic coagulation proteins at the cost of extrahepatic proteins like osteocalcin, MGP and endothelial protein S [3, 26, 62]. We have previously demonstrated that COVID-19 patients with extrahepatic vitamin K deficiency shown by elevated levels of dp-ucMGP, had normal levels of activated hepatic coagulation protein factor II [26]. As there are no indications that additional vitamin K supplementation in persons with fully activated hepatic coagulation proteins leads to a prothrombotic state, vitamin K supplementation in COVID-19 patients will likely selectively affect carboxylation of extrahepatic proteins.

The function of vitamin K is conserved between different species and toxicity studies in animal models have shown no adverse effects of high doses [27].

e. Analysis of potential effect

The safety and tolerability of vitamin K2 MK-7 supplementation in COVID-19 patients will be extensively assessed in this study. Supplementation will only be provided during hospital admission, where patients will be seen by a physician daily, and complete laboratory assessment will be performed three times a week.

The effects of vitamin K on carboxylation status of both hepatic and extrahepatic proteins will be assess by measuring PIVKA-II and dp-ucMGP, respectively.

f. Pharmacokinetic considerations

Vitamin K2 MK-7 has a longer half-life than most other forms of vitamin K, which has been extensively studied in healthy humans. Unlike other fat-soluble vitamins, both animal toxicity studies and observations from neonates administered high doses of vitamin K as prophylaxis

have shown no evidence of accumulation of vitamin K in tissues or formation of toxic metabolites.

g. Study population

Patients with COVID-19 requiring supplemental oxygen will be included in the study. Patients who are already admitted to the Intensive Care will not be included, however, admission to the ICU during the study does not require discontinuation of treatment. As additional protection against thrombotic complications all patients will be administered either prophylactic or therapeutic dosages of heparin-like anticoagulants. Pregnant women will not be included into the study.

h. Interaction with other products

The only known interaction risk with vitamin K are vitamin K antagonist drugs. Patients may not use VKAs during the study. If a patient is using VKAs at admission and wishes to participate in the study they must be switched to therapeutic dosages of heparin-based anticoagulants. In this case vitamin K2 MK-7/placebo treatment may only be initiated after the patient has received at least one dose of heparin-based anticoagulant.

i. Predictability of effect

Dp-ucMGP and PIVKA-II will be used to assess the effect of vitamin K2 MK-7 supplementation on extra- and intrahepatic vitamin K status, respectively. Both dp-ucMGP and PIVKA-II are well established robust biomarkers.

j. Can effects be managed?

There are no expected adverse effects.

13.2 Synthesis

Vitamin K supplementation has been extensively studied in healthy volunteers and several diseases including osteoporosis, cardiovascular disease and hemodialysis patients. These studies have shown no adverse effects. Toxicity studies in animals as well as observations in newborns given very high doses of vitamin K have shown no tolerability problems, no unwanted effects and no accumulation of toxic metabolites.

Vitamin K2 MK-7 is approved for sale in the European Union and freely available in The Netherlands. In a 2017 analysis the EFSA was unable to set any upper limit for the toxicity of any form of vitamin K. The current study aims to evaluate the effect of vitamin K2 MK-7 supplementation at a dose of 1000µg per day, which is similar to dosages of around 900µg

per day and 2000µg three times a week previously studied in healthy volunteers and hemodialysis patients, respectively.

Based on all available data, we were unable to predict any risks to the health of COVID-19 patients taking vitamin K2 MK-7 supplementation who are not on VKAs. During the study we will further minimize any unforeseen risks by only providing supplementation during hospital admission, when adverse events will be monitored daily and full hematology and clinical chemistry panels will be performed three times per week.

Because of its well-known role in coagulation extra attention will be paid to the development of thromboembolic complications and all patients will receive at least prophylactic dosages of heparin-based anticoagulants. Both measures are already part of standard protocol due to the high incidence of thrombotic complications in the normal progression of COVID-19.

14. REFERENCES

1. Zhou, F., et al., *Clinical course and risk factors for mortality of adult inpatients with COVID-19 in Wuhan, China: a retrospective cohort study.* Lancet, 2020. **395**(10229): p. 1054-1062.
2. Cui, S., et al., *Prevalence of venous thromboembolism in patients with severe novel coronavirus pneumonia.* J Thromb Haemost, 2020.
3. Fair, D.S., R.A. Marlar, and E.G. Levin, *Human endothelial cells synthesize protein S.* Blood, 1986. **67**(4): p. 1168-71.
4. Luo, G., et al., *Spontaneous calcification of arteries and cartilage in mice lacking matrix GLA protein.* Nature, 1997. **386**(6620): p. 78-81.
5. Chatrou, M.L., et al., *Vascular calcification: the price to pay for anticoagulation therapy with vitamin K-antagonists.* Blood Rev, 2012. **26**(4): p. 155-66.
6. Fraser, J.D. and P.A. Price, *Lung, heart, and kidney express high levels of mRNA for the vitamin K-dependent matrix Gla protein. Implications for the possible functions of matrix Gla protein and for the tissue distribution of the gamma-carboxylase.* J Biol Chem, 1988. **263**(23): p. 11033-6.
7. Price, P.A., J.R. Buckley, and M.K. Williamson, *The amino bisphosphonate ibandronate prevents vitamin D toxicity and inhibits vitamin D-induced calcification of arteries, cartilage, lungs and kidneys in rats.* J Nutr, 2001. **131**(11): p. 2910-5.
8. Rucker, R.B., *Calcium binding to elastin.* Adv Exp Med Biol, 1974. **48**(0): p. 185-209.
9. Basalyga, D.M., et al., *Elastin degradation and calcification in an abdominal aorta injury model: role of matrix metalloproteinases.* Circulation, 2004. **110**(22): p. 3480-7.
10. Bouvet, C., et al., *Sequential activation of matrix metalloproteinase 9 and transforming growth factor beta in arterial elastocalcinosis.* Arterioscler Thromb Vasc Biol, 2008. **28**(5): p. 856-62.
11. Shearer, M.J. and P. Newman, *Recent trends in the metabolism and cell biology of vitamin K with special reference to vitamin K cycling and MK-4 biosynthesis.* J Lipid Res, 2014. **55**(3): p. 345-62.
12. Alperin, J.B., *Coagulopathy caused by vitamin K deficiency in critically ill, hospitalized patients.* JAMA, 1987. **258**(14): p. 1916-9.
13. Usui, Y., et al., *Vitamin K concentrations in the plasma and liver of surgical patients.* Am J Clin Nutr, 1990. **51**(5): p. 846-52.
14. Ames, B.N., *Low micronutrient intake may accelerate the degenerative diseases of aging through allocation of scarce micronutrients by triage.* Proc Natl Acad Sci U S A, 2006. **103**(47): p. 17589-94.

15. Booth, S.L., et al., *Dietary phylloquinone depletion and repletion in older women.* J Nutr, 2003. **133**(8): p. 2565-9.
16. Schurgers, L.J., et al., *Effect of vitamin K intake on the stability of oral anticoagulant treatment: dose-response relationships in healthy subjects.* Blood, 2004. **104**(9): p. 2682-9.
17. Nigwekar, S.U., R. Thadhani, and V.M. Brandenburg, *Calciphylaxis.* N Engl J Med, 2018. **378**(18): p. 1704-1714.
18. Azoulay, L., et al., *Initiation of warfarin in patients with atrial fibrillation: early effects on ischaemic strokes.* Eur Heart J, 2014. **35**(28): p. 1881-7.
19. Shea, M.K. and S.L. Booth, *Concepts and Controversies in Evaluating Vitamin K Status in Population-Based Studies.* Nutrients, 2016. **8**(1).
20. Brandenburg, V.M., et al., *Slower Progress of Aortic Valve Calcification With Vitamin K Supplementation: Results From a Prospective Interventional Proof-of-Concept Study.* Circulation, 2017. **135**(21): p. 2081-2083.
21. Knapen, M.H., et al., *Menaquinone-7 supplementation improves arterial stiffness in healthy postmenopausal women. A double-blind randomised clinical trial.* Thromb Haemost, 2015. **113**(5): p. 1135-44.
22. Knapen, M.H., et al., *Three-year low-dose menaquinone-7 supplementation helps decrease bone loss in healthy postmenopausal women.* Osteoporos Int, 2013. **24**(9): p. 2499-507.
23. Janssen, R. and C. Vermeer, *Vitamin K deficit and elastolysis theory in pulmonary elasto-degenerative diseases.* Med Hypotheses, 2017. **108**: p. 38-41.
24. Cranenburg, E.C., et al., *Characterisation and potential diagnostic value of circulating matrix Gla protein (MGP) species.* Thromb Haemost, 2010. **104**(4): p. 811-22.
25. Akbulut, A.C., et al., *Vitamin K2 Needs an RDI Separate from Vitamin K1.* Nutrients, 2020. **12**(6).
26. Dofferhoff, A.S.M., et al., *Reduced vitamin K status as a potentially modifiable risk factor of severe COVID-19.* Clin Infect Dis, 2020.
27. Efsa Panel on Dietetic Products, N., et al., *Dietary reference values for vitamin K.* EFSA J, 2017. **15**(5): p. e04780.
28. Berkner, K.L., *Vitamin K-dependent carboxylation.* Vitam Horm, 2008. **78**: p. 131-56.
29. Theuwissen, E., E. Smit, and C. Vermeer, *The role of vitamin K in soft-tissue calcification.* Adv Nutr, 2012. **3**(2): p. 166-73.
30. Vermeer, C. and M. Ulrich, *Vitamin K-dependent carboxylase in horse liver, spleen and kidney.* Thromb Res, 1982. **28**(2): p. 171-7.
31. de Boer-van den Berg, M.A., C.P. Verstijnen, and C. Vermeer, *Vitamin K-dependent carboxylase in skin.* J Invest Dermatol, 1986. **87**(3): p. 377-80.

32. Cranenburg, E.C., L.J. Schurgers, and C. Vermeer, *Vitamin K: the coagulation vitamin that became omnipotent.* Thromb Haemost, 2007. **98**(1): p. 120-5.
33. Willems, B.A., et al., *The realm of vitamin K dependent proteins: shifting from coagulation toward calcification.* Mol Nutr Food Res, 2014. **58**(8): p. 1620-35.
34. Gheduzzi, D., et al., *Matrix Gla protein is involved in elastic fiber calcification in the dermis of pseudoxanthoma elasticum patients.* Lab Invest, 2007. **87**(10): p. 998-1008.
35. Boraldi, F., et al., *Matrix gla protein and alkaline phosphatase are differently modulated in human dermal fibroblasts from PXE patients and controls.* J Invest Dermatol, 2013. **133**(4): p. 946-54.
36. Schurgers, L.J., et al., *Post-translational modifications regulate matrix Gla protein function: importance for inhibition of vascular smooth muscle cell calcification.* J Thromb Haemost, 2007. **5**(12): p. 2503-11.
37. Price, P.A., D. Toroian, and J.E. Lim, *Mineralization by inhibitor exclusion: the calcification of collagen with fetuin.* J Biol Chem, 2009. **284**(25): p. 17092-101.
38. Pivin, E., et al., *Inactive Matrix Gla-Protein Is Associated With Arterial Stiffness in an Adult Population-Based Study.* Hypertension, 2015. **66**(1): p. 85-92.
39. Spronk, H.M., et al., *Tissue-specific utilization of menaquinone-4 results in the prevention of arterial calcification in warfarin-treated rats.* J Vasc Res, 2003. **40**(6): p. 531-7.
40. Rennenberg, R.J., et al., *Chronic coumarin treatment is associated with increased extracoronary arterial calcification in humans.* Blood, 2010. **115**(24): p. 5121-3.
41. H. Molitor, H.J.R., *Oral and parenteral toxicity of vitamin K1, phthicol and 2-methyl-1,4-naphthoquinone.* Proceedings of the Society for Experimental Biology and Medicine, 1940. **43**: p. 125-128.
42. Pucaj, K., et al., *Safety and toxicological evaluation of a synthetic vitamin K2, menaquinone-7.* Toxicol Mech Methods, 2011. **21**(7): p. 520-32.
43. Ravishankar, B., et al., *Safety assessment of menaquinone-7 for use in human nutrition.* J Food Drug Anal, 2015. **23**(1): p. 99-108.
44. Mitsui, N., *Effect of Natto including Bacillus subtilis K-2 (spore) on defecation and fecal microbiota and safety of excessive ingestion in healthy volunteers.* Jpn Pharmacol Ther, 2006. **34**(1): p. 135-148.
45. Sumi, H., *Accumulation of vitamin K (Menaquinone-7) in plasma after ingestion of natto and natto bacilli (B. subtilis natto).* Food Science Technology Research, 1999. **5**(1): p. 48-50.
46. Tsukamoto, Y., et al., *Intake of fermented soybean (natto) increases circulating vitamin K2 (menaquinone-7) and gamma-carboxylated osteocalcin concentration in normal individuals.* J Bone Miner Metab, 2000. **18**(4): p. 216-22.

47. Yamaguchi, M., et al., *Prolonged intake of fermented soybean (natto) diets containing vitamin K2 (menaquinone-7) prevents bone loss in ovariectomized rats.* J Bone Miner Metab, 2000. **18**(2): p. 71-6.
48. Homma, K., et al., *Treatment of natto, a fermented soybean preparation, to prevent excessive plasma vitamin K concentrations in patients taking warfarin.* J Nutr Sci Vitaminol (Tokyo), 2006. **52**(5): p. 297-301.
49. Asakura, H., et al., *Vitamin K administration to elderly patients with osteoporosis induces no hemostatic activation, even in those with suspected vitamin K deficiency.* Osteoporos Int, 2001. **12**(12): p. 996-1000.
50. De Vriese, A.S., et al., *Multicenter Randomized Controlled Trial of Vitamin K Antagonist Replacement by Rivaroxaban with or without Vitamin K2 in Hemodialysis Patients with Atrial Fibrillation: the Valkyrie Study.* J Am Soc Nephrol, 2020. **31**(1): p. 186-196.
51. Lindholt, J.S., et al., *Effects of menaquinone-7 supplementation in patients with aortic valve calcification: study protocol for a randomised controlled trial.* BMJ Open, 2018. **8**(8): p. e022019.
52. Ansumana, R., et al., *Ebola in Freetown area, Sierra Leone--a case study of 581 patients.* N Engl J Med, 2015. **372**(6): p. 587-8.
53. Caluwe, R., et al., *Vitamin K2 supplementation in haemodialysis patients: a randomized dose-finding study.* Nephrol Dial Transplant, 2014. **29**(7): p. 1385-90.
54. Hirsh, J., et al., *Mechanism of action and pharmacology of unfractionated heparin.* Arterioscler Thromb Vasc Biol, 2001. **21**(7): p. 1094-6.
55. Castellucci, L.A., et al., *Clinical and safety outcomes associated with treatment of acute venous thromboembolism: a systematic review and meta-analysis.* JAMA, 2014. **312**(11): p. 1122-35.
56. Felder, S., et al., *Prolonged thromboprophylaxis with low molecular weight heparin for abdominal or pelvic surgery.* Cochrane Database Syst Rev, 2019. **3**: p. CD004318.
57. Ma, S., Y.Y. Lin, and G.M. Turino, *Measurements of desmosine and isodesmosine by mass spectrometry in COPD.* Chest, 2007. **131**(5): p. 1363-71.
58. Ma, S., et al., *Stable deuterium internal standard for the isotope-dilution LC-MS/MS analysis of elastin degradation.* Anal Biochem, 2013. **440**(2): p. 158-65.
59. Araki, S. and A. Shirahata, *Vitamin K Deficiency Bleeding in Infancy.* Nutrients, 2020. **12**(3).
60. Lemke, G., *Biology of the TAM receptors.* Cold Spring Harb Perspect Biol, 2013. **5**(11): p. a009076.
61. Lemke, G. and G.J. Silverman, *Blood clots and TAM receptor signalling in COVID-19 pathogenesis.* Nat Rev Immunol, 2020. **20**(7): p. 395-396.

62. McCann, J.C. and B.N. Ames, *Vitamin K, an example of triage theory: is micronutrient inadequacy linked to diseases of aging?* Am J Clin Nutr, 2009. **90**(4): p. 889-907.
